# SGK3 promotes vascular calcification via Pit-1 in chronic kidney disease

**DOI:** 10.7150/thno.87317

**Published:** 2024-01-01

**Authors:** Qing-Qing Dong, Yu-Chi Tu, Pan Gao, Qian-Qian Liao, Peng Zhou, Hui Zhang, Hua-Pan Shu, Lu-Lu Sun, Li Feng, Li-Jun Yao

**Affiliations:** 1 Department of Nephrology, Union Hospital, Tongji Medical College, Huazhong University of Science and Technology, Wuhan, China.; 2 Department of Nephrology, The Second Affiliated Hospital, Chongqing Medical University, Chongqing, China.; 3 Department of Ultrasonography, Union Hospital, Tongji Medical College, Huazhong University of Science and Technology, Wuhan, China.; 4 Department of Vascular Surgery, Union Hospital, Tongji Medical College, Huazhong University of Science and Technology, Wuhan, China.; 5 Department of Nephrology, Wuhan Fourth Hospital, Wuhan, China.

**Keywords:** SGK3, vascular calcification, Pit-1, ubiquitin-proteasome pathway, chronic kidney disease

## Abstract

**Rationale:** Vascular calcification (VC) is a life-threatening complication in patients with chronic kidney disease (CKD) caused mainly by hyperphosphatemia. However, the regulation of VC remains unclear despite extensive research. Although serum- and glucocorticoid-induced kinase 3 (SGK3) regulate the sodium-dependent phosphate cotransporters in the intestine and kidney, its effect on VC in CKD remains unknown. Additionally, type III sodium-dependent phosphate cotransporter-1 (Pit-1) plays a significant role in VC development induced by high phosphate in vascular smooth muscle cells (VSMCs). However, it remains unclear whether SGK3 regulates Pit-1 and how exactly SGK3 promotes VC in CKD via Pit-1 at the molecular level. Thus, we investigated the role of SGK3 in the certified outflow vein of arteriovenous fistulas (AVF) and aortas of uremic mice.

**Methods and Results:** In our study, using uremic mice, we observed a significant upregulation of SGK3 and calcium deposition in certified outflow veins of the AVF and aortas, and the increase expression of SGK3 was positively correlated with calcium deposition in uremic aortas. *In vitro*, the downregulation of SGK3 reversed VSMCs calcification and phenotype switching induced by high phosphate. Mechanistically, SGK3 activation enhanced the mRNA transcription of Pit-1 through NF-κB, downregulated the ubiquitin-proteasome mediated degradation of Pit-1 via inhibiting the activity of neural precursor cells expressing developmentally downregulated protein 4 subtype 2 (Nedd4-2), an E3 ubiquitin ligase. Moreover, under high phosphate stimulation, the enhanced phosphate uptake induced by SGK3 activation was independent of the increased protein expression of Pit-1. Our co-immunoprecipitation and *in vitro* kinase assays confirmed that SGK3 interacts with Pit-1 through Thr468 in loop7, leading to enhanced phosphate uptake.

**Conclusion:** Thus, it is justifiable to conclude that SGK3 promotes VC in CKD by enhancing the expression and activities of Pit-1, which indicate that SGK3 could be a therapeutic target for VC in CKD.

## Introduction

Vascular calcification (VC) is the pathological deposition of minerals in the form of calcium salts and phosphates in vascular tissues 1. This condition is due to elevated serum phosphate levels and calcium phosphate production (Ca/P), which involve many key regulators of bone formation and structural proteins 2. VC is highly prevalent in patients with end-stage renal diseases (ESRD), particularly those on dialysis. Studies have shown that the participants with advanced CKD were more likely to have cardiovascular disease 3, and that coronary artery calcification in dialysis patients increases by an average of 15% annually, which may be a leading cause of death from cardiovascular events 4. Additionally, approximately 32.9% of patients with CKD exhibit abdominal aortic calcification, and the risk of cardiovascular events is significantly higher in patients with CKD and VC than in those without VC 3.

Vascular smooth muscle cells (VSMCs) can undergo phenotypic switching from a contractile to a synthetic phenotype in vascular diseases such as renal failure, diabetes, coronary artery disease 5. Under various pathological conditions, including elevated levels of plasma uremic toxins, indoxyl sulfate, mineral dysregulation, and hyperphosphatemia, VSMCs can differentiate into chondrocytes and osteoblasts, thereby promoting VC. This process is characterized by an increase in the expression of osteogenic proteins, including runt-related transcription factor 2 (RUNX2), and bone morphogenetic protein 2 (BMP2), as well as a decrease in the expression of the contractile protein alpha-smooth muscle actin (α-SMA) in VSMCs 6, 7. There are three types of NaPi cotransporters in mammal. The type I cotransporters are involved in organic anion transport, which is expressed in kidney, livers, stomach and intestine. The type II cotransporters, including NaPi2b, predominantly expressed in kidney and intestinal epithelium tissues rather than in vascular smooth cells. The main NaPi cotransporters on VSMCs were type III NaPi cotransporters, Pit-1 and Pit-2 8. Pit-1 is the major phosphate transport protein found in VSMCs; it enhances phosphate uptake and BMP-2 and RUNX2 expression in response to elevated phosphate levels 9. As such, Pit-1 plays an important role in VC and VSMCs differentiation 10, 11. However, the precise molecular mechanisms by which Pit-1 contributes to VC have not yet been fully elucidated.

Serum- and glucocorticoid-induced kinase (SGK), is a type of serine/threonine protein kinase that belongs to the AGC family and is found in various mammalian tissues and organs, including VSMCs 12. The SGK family consists of three highly homologous mammalian isoforms, SGK1, SGK2, and SGK3, each encoded by a different gene 13. Studies have demonstrated that SGK1 can activate VC and neointimal formation in vein grafts and up to 80% of the amino acid sequences of the catalytic domains of SGK3 are homologous to those of SGK1 14, 15. However, the role of SGK3 in VC has not yet been elucidated. Recent studies have shown that SGK3 regulates NaPi2b, a critical intracellular intestinal phosphate transporter 16. SGK3 knockout mice exhibited increased urinary phosphate excretion and decreased plasma calcitriol and fibroblast growth factor 23 (FGF23) concentrations 17, all of which play a significant role in VSMCs phenotype switching. Therefore, we speculated that SGK3 may regulate the phosphate transporter Pit-1 in VSMCs, and that SGK3 mediated Pit-1 may be associated with VSMCs phenotype switching and VC.

Thus, our study aimed to clarify the molecular mechanisms underlying how SGK3 triggers Pit-1 to facilitate VC in patients with CKD-induced hyperphosphatemia. By doing so, we aimed to identify potential therapeutic targets for VC in CKD.

## Materials and Methods

### Reagents and antibodies

Polyclonal rabbit anti-neural precursor cells expressing developmentally downregulated protein 4 subtype 2 (Nedd4-2) (1:1000), anti-SGK3 (1:1000) and anti-phospho-Nedd4-2 (Ser342) (1:1000) antibodies were purchased from Cell Signaling Technology (Beverly, MA, USA). Polyclonal rabbit anti-SGK3 antibody for immunohistochemistry was purchased from Abnova (Taipei, Taiwan, China). Monoclonal mouse anti-ubiquitin (Ub) (1:800) antibody was purchased from Covance Inc. (UT, USA). Monoclonal mouse Anti-Phosphothreonine (1:1000) and polyclonal rabbit Anti-Phosphoserine (1:1000) antibodies were purchased from Sigma-Aldrich (St. Louis, MO, USA). Polyclonal rabbit anti-Pit-1 (1:800), monoclonal mouse anti-GAPDH (1:5000), horseradish peroxide-conjugated anti-rabbit and anti-mouse secondary antibodies (1:5000) were purchased from Proteintech (Wuhan, China).

The pcDNA3/mNedd4-2 and pcDNA3.1/mSGK3-S486D plasmids were gifts from David Pearce (Departments of Medicine, and Molecular and Cellular Pharmacology, University of California, San Francisco, CA, 94107-2140, USA) 18. The Pit-1-Myc-DDK plasmid was purchased from Origene Technologies, Inc. (Rockville, MD, USA). The plasmid of the mouse Flag-tagged WT ubiquitin (Ub) was provided by Dr. Qiuhong Duan (Department of Biochemistry and Molecular Biology, Tongji Medical College, Huazhong University of Science & Technology, Wuhan, China). Recombinant lentivirus SGK3-shRNA was designed and generated by Genechem (Shanghai, China) as previously described 19. The SGK3 siRNA and Nedd4-2 siRNA were constructed by RiboBio (Guangzhou, China). β-glycerophosphate was purchased from Millipore-Sigma (St Louis, MO, USA). SGK3-PROTAC1 was purchased from MedChemExpress (Shanghai, China). The NF-κB inhibitor BAY11-7085 and MG132 were purchased from Selleck (Shanghai, China). Protein A+G Agarose for co-immunoprecipitation (co-IP) assay was purchased from Beyotime Biotechnology (Shanghai, China). Cocktail and phosphatase inhibitors were purchased from Roche Applied Science (Indianapolis, IN, USA).

### Human samples

Uremic serum was collected from 20 non-dialysis CKD stage 5 patients (18-70 years old), and normal serum from 20 healthy individuals of similar age and sex was used as controls. The exclusion criteria included pregnancy, diabetes, immunologic or tumor related disease history, acute inflammatory or infectious episode, hepatitis B, hepatitis C, or human immunodeficiency virus (HIV) infection. After centrifugation aliquots of individual serum samples were tested for creatinine, urea nitrogen, calcium and phosphate by standard laboratory methods. The rest uremic serum or control serum was pooled, respectively, then filtered through 0.22 μm membranes, and stored at -80 °C until use. Patients and healthy individuals were recruited at the Union Hospital, Tongji Medical College, Huazhong University of Science and Technology (Wuhan, China) after approval by the Ethics Committee of Wuhan Union Hospital (Approval [2021] No. 0719-01) and the study was conducted in accordance with the standards set by the latest revision of the Declaration of Helsinki. Written informed consent was secured from all subjects or their legal guardians, and followed the guidelines from ICMJE on Protection of Research Participants. Characteristics of the pooled serums are shown in Table S1.

### Murine models of CKD and AVF

All animal experiments were conducted in accordance with institutional guidelines and an approved protocol of Tongji Medical College, Huazhong University of Science and Technology (Approval No. S2433). Female DBA2 mice (20-25 g, 12-13 wk old) and male C57BL/6J mice (20-25 g, 8-10 wk old) were purchased from SPF Biotechnology Co., Ltd. (Beijing, China) and bred in a specific pathogen-free barrier facility. The DBA2 mice have an inherent susceptibility to high-phosphate diet-triggered calcification, in contrast to C57BL/6 that are resistant 20. Female DBA2 mice were used since they showed higher susceptibility to calcification than males 21.

The mice were randomly divided into sham operation and 5/6 nephrectomy (CKD) groups. A sham operation was performed on the sham-operated mice. A mouse model of CKD was induced by 5/6th partial nephrectomy as described previously in two surgical sessions 22. Mice were anesthetized with a pentobarbital (50 mg/kg) by intraperitoneal injection. Firstly, through a flank incision, the left kidney was decapsulated to avoid ureter and adrenal damage, and the left kidney mass was reduced to one third. One week later, the right nephrectomy was performed via a flank incision. After one week of recovery, the mice were fed a high phosphate (1.8%) diet (Dyets Inc., USA). The serum of mice was collected after 4 weeks of dietary intervention, and plasma calcium, plasma phosphate, blood urea nitrogen and creatinine levels were measured by using the respective kits (Nanjing Jiancheng Bioengineering Institute, Nanjing, China).

A mouse model of arteriovenous fistula (AVF) was established by an end-vein to anastomosis of the right internal jugular vein and the right common carotid artery 4 weeks after right nephrectomy 23. The right internal jugular vein and the right common carotid artery were carefully isolated using a dissecting microscope in mouse. The proximal end was clamped and the distal end was ligated of internal jugular vein. The proximal end of the common carotid artery was clamped. An anastomosis was created between the vein and artery using 11-0 nylon suture with an interrupted stitch. After unclasping, flow through the fistula was confirmed. At 4 weeks after placement of the AVF, mice were euthanized with an overdose pentobarbital (100 mg/kg) injected intraperitoneally. The outflow vein of the AVF, aorta and kidneys were collected for analysis of protein expression or morphological changes.

### Cell culture

The mouse VSMCs were gifts from the Department of Cardiology, Union Hospital, Tongji Medical College, Huazhong University of Science and Technology. The human VSMCs was purchased from the Shanghai Cell Bank of the BLUEFBIO. The HEK293T were gifts from the Regeneration Laboratory of Wuhan Union Medical College Hospital.

VSMCs and HEK293T cells were cultured in Dulbecco's modified Eagle's medium (HyClone, USA) supplemented in the presence of 10% fetal bovine serum (ScienCell, San Diego, CA, USA) and 1% penicillin/streptomycin at 37 °C humidified incubator containing 5% CO_2_. The cells were cultured in 6-well plates at a density of 1.5×10^5^ cells per well 24 h prior to the experiments. For elevated phosphate conditions, VSMCs were cultured in 3 mM NaH_2_PO_4_/Na_2_HPO_4_, calcification media (CM) (10 mM β-glycerophosphate, 0.25 mM L-ascorbic acid, and 10^-8^ mM dexamethasone), or 20% uremic serum. Cells were used for 2 h (phosphate uptake assays), 24 h (RT-qPCR), 48 h (Western blotting, ubiquitination assay, co-IP assays), or 7 days (Alizarin Red S staining, calcium Deposition) after high phosphate conditions. VSMCs and HEK293T cells were transiently transfected with various plasmids using Lipofectamine 2000 Reagent, according to the manufacturer's instructions (Thermo Fisher Scientific). VSMCs were treated either with different concentrations of SGK3-PROTAC1 (1 μM, 2.5 μM) for 24 h, or with 2.5 μM SGK3-PROTAC1 for 8 h, 12 h and 24 h.

### Cell transfection

Mouse VSMCs and HEK293T cells were transfected with various plasmids for 24-48 h by Lipofectamine 2000 Reagent according to the manufacturer's instructions (Invitrogen, Carlsbad, CA, United States). The VSMCs were transfected with SGK3 siRNA for 48 h or SGK3 shRNA lentivirus for 72 h, and then the cell lysates were used for immunoblotting analysis as previously described 19, 24.

### Histochemistry and immunohistochemistry

The vascular tissues were fixed in 4% paraformaldehyde and embedded for histochemistry and immunohistochemistry studies. Immunohistochemistry staining proceeded and analyzed essentially as previously described 24. In short, slides were incubated with polyclonal rabbit anti-SGK3 antibody overnight at 4 °C, followed by horseradish peroxidase-coupled secondary antibody for 30 min at room temperature. The bound antibody was visualized using a DAB kit. All images were acquired using light microscopy. Positive expression for SGK3 was semi-quantitative analyzed using the Image J software in at least 2 sections of each animal and at least 5 images taken in different areas within the samples.

### Immunofluorescence

Mouse VSMCs were washed with PBS and fixed with 4% paraformaldehyde for 10 min at room temperature. Then, cells were permeabilized with 0.1% Triton X-100 for 15 min at room temperature and incubated with polyclonal anti-NF-κB antibody overnight at 4 °C. The cells were stained with Fluorescein (FITC)-conjugated Affinipure Goat Anti-Rabbit IgG (H+L) (Proteintech, Wuhan, China) and the nuclei were stained with 4, 6-diamidino-2-phenylindole (DAPI) for 5 min. All sections were observed under a fluorescent microscope (Nikon, Tokyo, Japan).

### Phosphate uptake assays

According to research reports that phosphate content of the cell lysate was measured using the Pi Assay Kit (Sigma-Aldrich) 25, 26. After 2 h of culture, cells were washed with PBS without Ca^2+^ and Mg^2+^, and solubilized with 1% Triton X-100 for 1.5 h, and cell lysates were assayed for phosphate using the Pi Assay Kit. The protein content of samples was measured using a BCA protein assay kit (Beyotime Biotechnology, Sanghai, China). Intracellular phosphate concentration was normalized to protein content expressed as μmol/mg protein.

### Alizarin Red S staining

Mouse or human VSMCs were cultured in 3 mM NaH_2_PO_4_/Na_2_HPO_4_, calcification media (CM), or 20% uremic serum for 7 days, respectively, and the medium was changed every other day. Cells were washed with PBS without Ca^2+^ and Mg^2+^, and fixed with 4% paraformaldehyde for 10 min at room temperature. Then, cells were washed with PBS and stained with 2% Alizarin Red S for 10 min. Next, excessive dye was removed by several washes, and the extracellular calcium deposition was stained in red color.

Vascular tissues were prepared as 6 μm serial sections for Alizarin Red S staining. After deparaffinization and hydration, tissues were stained with 2% Alizarin red S solution for 30 min at room temperature. All images were obtained using light microscopy.

### Measurement of calcium deposition

The cells were washed twice with PBS without Ca^2+^ and Mg^2+^, and decalcified with 0.6 mol/L HCl overnight at 37 °C. The calcium content in the HCl supernatant was subjected to colourimetric analysis using a calcium assay kit (Nanjing Jiancheng Bioengineering Institute, Nanjing, China). After decalcification, cells were washed with PBS and solubilized with a solution of 0.1 mol/L NaOH and 0.1% SDS, and the protein content of samples was measured using a BCA protein assay kit (Beyotime Biotechnology, Sanghai, China). The calcium content was normalized to protein content.

### Immunoblotting analysis

Vascular tissues and cells were lysed in RIPA buffer (Beyotime Biotechnology, Shanghai, China) to prepare whole proteins. Equal amounts of proteins were separated by electrophoresis on sodium dodecyl sulfate-polyacrylamide gel electrophoresis (SDS-PAGE) gels and transferred onto polyvinylidene difluoride membrane. The membrane was incubated with the appropriate primary antibodies at 4 °C overnight, and protein signals were detected using the corresponding horseradish peroxidase-conjugated secondary antibodies for 1 h at room temperature. All experiments were performed in triplicate.

### Extraction of nuclear and cytoplasmic proteins

Nuclear and cytoplasmic proteins were isolated using the Nuclear and Cytoplasmic Protein Extraction Kit (Beyotime Biotechnology. Shanghai, China) according to the manufacturer's instructions.

### RNA extraction, reverse transcription, and quantitative Real-Time PCR

Total RNA was extracted from incubated cells using TRIzol reagent (Vazyme, Nanjing, China) and then reverse transcribed it into cDNA by PrimeScript RT Master Mix (Takara, Dalian, China) according to the manufacturer's instructions. The mRNA levels of the target genes were measured with the SYBR Premix Ex Taq (Takara, Dalian, China) on a StepOnePlusTM Real-Time PCR System. The results for were normalized using beta-actin mRNA as an internal control and analyzed by ΔΔCt. All the experiment was performed in triplicate. The primers for mouse and human SGK3, Pit-1, and beta-actin were designed based on the GenBank accession numbers (SGK3: NM_133220, NM_013257, Pit-1: NM_015747). The sequences of the primer pairs used in qRT-PCR are as follows: mouse SGK3 forward: AGAAAACAGCCCTATGACAACAC, reverse: AGCAACATCTCGGCAGTAAAA; mouse Pit-1 forward: ATGAGTTGCCAATCTTTCACCTC, reverse: GCTGTGGACATCACGTTGGT; mouse beta-actin forward: TGCTGTCCCTGTATGCCTCTG, reverse: TGATGTCACGCACGATTTCC; human SGK3 forward: TGAGGCCAGGAGTGAGTCTT, reverse: CTGCCTCCAATACACTGGCA; human beta-actin forward: CACCATTGGCAATGAGCGGTTC, reverse: AGGTCTTTGCGGATGTCCACGT.

### Ubiquitination assay

To prevent proteasomal degradation of ubiquitinated Pit-1, cells were treated with MG132 (10 μM) for 8 h before harvest. Ubiquitination assays were performed as described 19. In brief, VSMCs were lysed in IP buffer (Beyotime Biotechnology. Shanghai, China) supplemented with PMSF, cocktail, and phosphatase inhibitors. The proteins (about 1 mg) were formulated into an equal volume system and added to the protein A+G agarose beads and anti-Pit-1 antibody overnight at 4 °C for co-immunoprecipitates. The samples were separated by SDS-polyacrylamide gel and immunoblotted with anti-ubiquitin (Ub) antibody.

### Co-immunoprecipitation (co-IP) assays

The cells were dissociated in IP lysis buffer supplemented with PMSF, cocktail, and phosphatase inhibitors. The proteins (about 1 mg) were incubated with the appropriate primary antibodies (anti-Pit-1 antibody or anti-SGK3 antibody) and protein A+G agarose beads overnight at 4 °C for co-IP. Then the samples were separated by SDS-polyacrylamide gel and immunoblotted with corresponding antibody.

### *In vitro* kinase assay

The SGK3 active kinase was purchased from Millipore Corporation (Billerica, MA). ATP and 10 × Kinase buffer were purchased from Cell Signaling Technology (Beverly, MA, USA). His-Pit-1-Loop7 was expressed in Escherichia coli bacteria. The inactive substrate (His-Pit-1-Loop7 3 μg) and the active SGK3 kinase (0.9 μg) were incubated together in a 30 μL reaction system (1 × Kinase buffer containing 10 mM ATP) at 37 °C for 2 h. The samples were added to 5 × SDS buffer, and then visualized by western blot analysis after resolving by SDS-PAGE.

### Statistical analysis

All experiments were conducted in triplicate. All data were expressed as Mean ± standard deviation (SD). Normality was tested with the Shapiro-Wilk test. All data presented in normal distribution. An F test (two groups) or Brown-Forsythe test (multiple groups) was used to determine difference in variances for t-test or ANOVA test, respectively. The unpaired Student's t-test or unpaired t-test with Welch's correction was used to analyze differences between two groups. Comparisons within more than 2 groups were performed by one-way or two-way ANOVA. A p-value less than 0.05 was considered statistically significant. Analyses were performed using Graph Pad Prism 8.0 software (Graph Pad Prism Software, La Jolla, CA, USA).

## Results

### SGK3 is upregulated in calcification of outflow vein in AVF and aorta from high phosphate diet administrated CKD Mice

**Figure 1:**
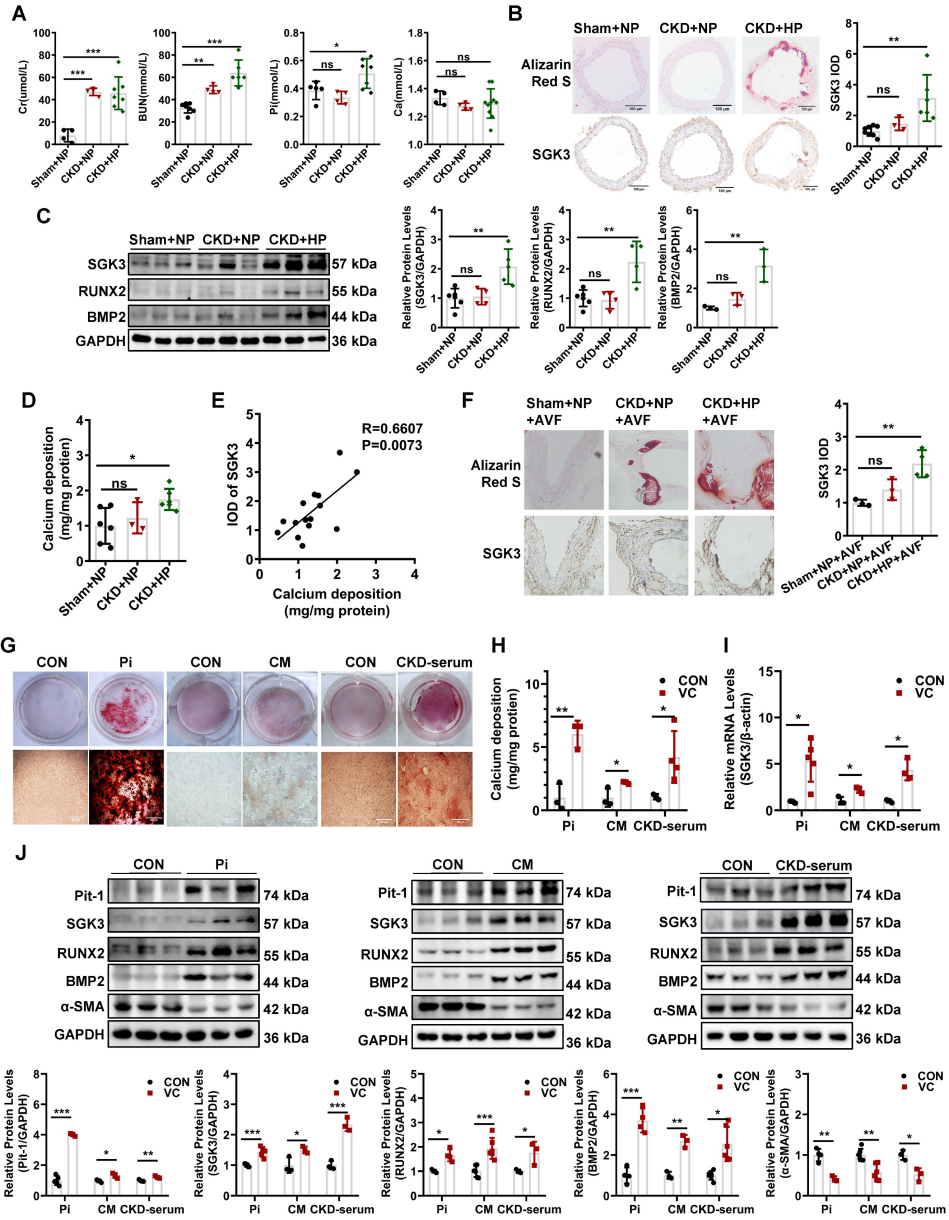
** Elevated protein expression of SGK3 in calcified VSMCs *in vivo* and *in vitro*.** Female DBA2 mice modeled with CKD by 5/6 nephrectomy and fed with a high-phosphate diet for 8 weeks after surgery.** A.** At the experimental end point, blood urea nitrogen (BUN), serum creatinine (Cr), calcium (Ca), and phosphate (Pi) were detected. **B.** Alizarin red S staining and representative immunohistochemical staining of SGK3 were detected in the aorta of CKD mice. **C.** The protein expression levels of SGK3, RUNX2 and BMP2 in the aorta of CKD mice, as assayed by immunoblotting. **D.** The total calcium content was detected in the aorta of CKD mice. **E.** There was a significantly positive correlation between SGK3 expression and calcium deposition in mice aorta. n=4-6 for each group. **F.** Alizarin red S staining and representative immunohistochemical staining of SGK3 in the AVF outflow vein of CKD mice. n=3 for each group. *P < 0.05, **P < 0.01, ***P < 0.001 vs. Sham+NP group. Mouse or human VSMCs treated with 3 mM Pi, calcification medium (CM), or 20% CKD-serum for 7 days. **G.** The calcium deposits in VSMCs was detected by Alizarin Red S staining.** H.** The calcium content in VSMCs was quantified by calcium assay kit. **I.** After 3 mM Pi, CM or 20% CKD-serum treatment for 24 h, the mRNA levels of SGK3 were detected with quantitative RT-PCR. **J.** Western blot and related semi-quantificated analysis of Pit-1, SGK3, BMP2, Runx2 and α-SMA in VSMCs with 3 mM Pi, CM or 20% CKD-serum for 7 days. *P < 0.05, **P < 0.01, ***P < 0.001 vs. control group.

To establish a VC mouse model, the DBA2 and C57BL/6J mice were subjected to 5/6 nephrectomy (5/6 Nx) and treated with a high-phosphate (HP) diet (CKD+HP) or a normal-phosphate (NP) diet (CKD+NP), as administration of an HP diet alone did not induce VC in mice 27, 28. Treatment with 5/6 Nx resulted in significantly increased serum creatinine and urea nitrogen levels both in DBA2 and in C57BL/6J mice (Figure 1A, Figure S1A). Serum levels of phosphate (Pi) in the CKD+HP group were much higher than those in the Sham+NP group (Figure 1A, Figure S1A). The positive areas of Alizarin Red S staining were significantly increased in aorta of CKD+HP group than those in the Sham+NP group of DBA2 mice (Figure 1B), while no positive stain could be detected on C57BL/6J mice (Figure S1B-C). Thus, we used DBA2 mice for our following experiment. The immunohistochemical analysis revealed that SGK3 expression levels were significantly higher in the calcified aorta of CKD+HP group than in those of Sham+NP group (Figure 1B). Western blot analysis revealed dramatically enhanced protein expression of SGK3, RUNX2, and BMP2 in the calcified aortas of CKD+HP group (Figure 1C). Compared with the Sham+NP group, the calcium content was significantly increased in the calcified aorta of CKD+HP group (Figure 1D). Furthermore, correlation analysis showed that SGK3 expression positively correlated with calcium deposition in the mouse aorta Figure 1E). Since the VC is one of the culprits of AVF malfunction patients with CKD, we constructed a CKD + HP + AVF calcification mouse model to study the relevance of SGK3 in the VC of CKD patients with AVF malfunction. Consistent with the calcified aorta, immunohistochemistry confirmed that SGK3 protein expression was significantly enhanced in the calcified outflow vein of AVFs from CKD mice (Figure 1F). These findings suggest the relevance of SGK3 in the VC with CKD.

### The expression of SGK3 is increased in cultured VSMCs treated with calcifying conditions

We explored the direct effects of several triggers of osteoblastic transdifferentiation and calcification of VSMCs in SGK3 expression. The mineralization of VSMCs was successfully (red signal) induced in high Pi (3 mM), or calcification medium (CM) containing 10 mmol/L β-glycerophosphate (10 mM β-glycerophosphate, 0.25 mM L-ascorbic acid, and 10^-8^ mM dexamethasone), or 20% pooled uremic serum from patients with stage 4 to 5 CKD (not on dialysis, ND-CKD) for 7 days as determined by Alizarin Red S staining (Figure 1G) and quantitative calcium measurement (Figure 1H). As illustrated in Figure 1I, treatment with high levels of Pi, CM, or 20% uremic serum significantly increased SGK3 mRNA expression in cultured VSMCs. Similarly, VSMCs treated with different calcification conditions significantly upregulated the protein levels of SGK3 and Pit-1 (Figure 1J, Figure S6A). This upregulation was accompanied by increased protein levels of the osteogenic markers BMP2 and RUNX2, and decreased protein levels of α-SMA (Figure 1J). Similarly, high phosphate induced enhanced phosphate uptake in cultured VSMCs (Figure S2A-C). Therefore, SGK3 may play a role in the development of VC in CKD.

### Inactivation of SGK3 mostly reverses high phosphate-induced calcification and phenotype switching of VSMCs

**Figure 2:**
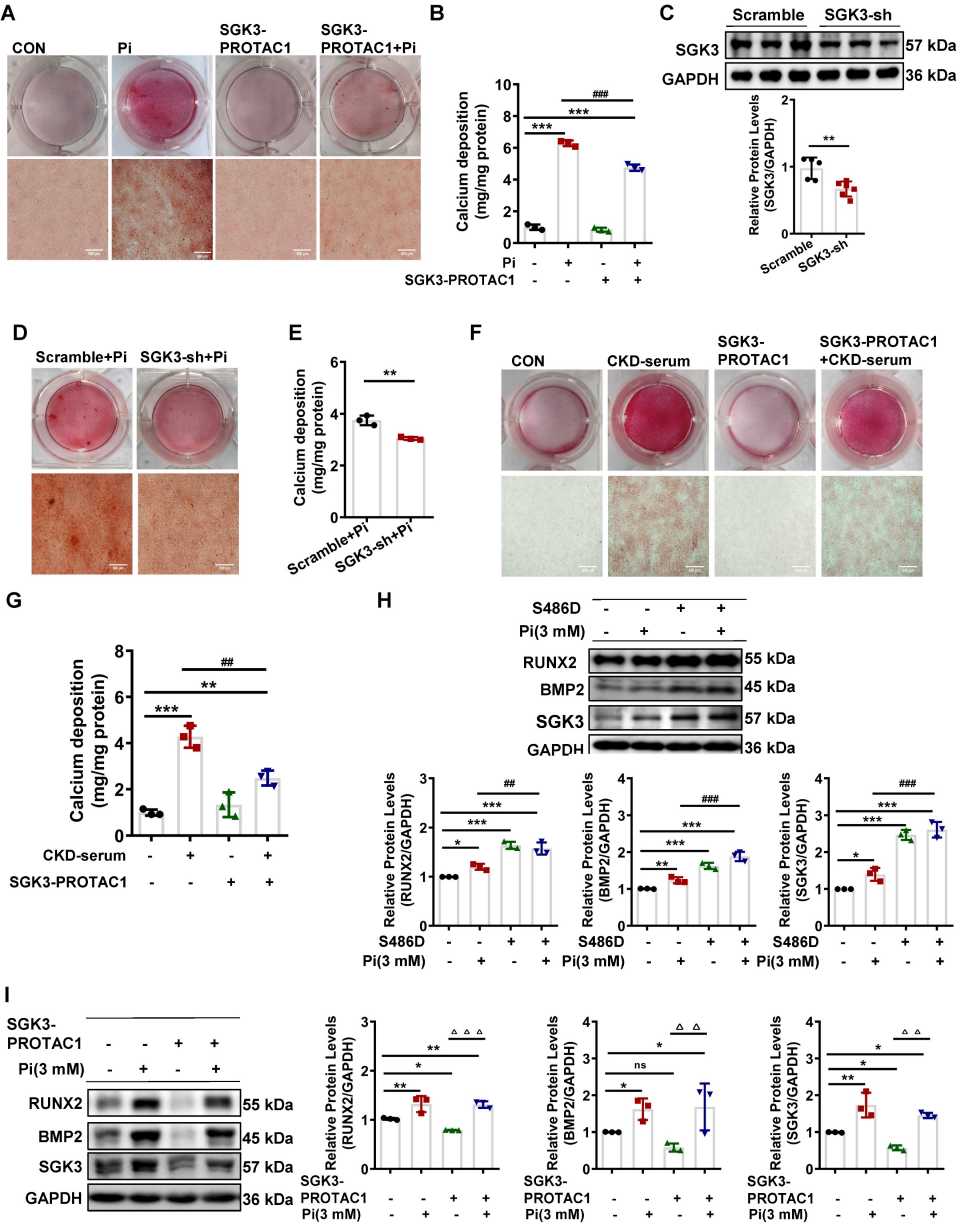
** Inhibition of SGK3 attenuated high phosphate-induced VSMCs calcification and phenotype switching *in vitro*. A-B.** Mouse VSMCs treated with 3 mM Pi and without or with additional treatment with 2.5 μmol/L SGK3 inhibitor (SGK3-PROTAC1) for 7 days. Representative original images showing Alizarin Red S staining (**A**). The cellular calcium content was evaluated quantitatively (**B**). ***P < 0.001 vs. control group; ^###^*P* < 0.001 vs. Pi group. **C.** Mouse VSMCs were infected with scramble or SGK3 shRNA (SGK3-sh) for 72 h. Western blot analysis of SGK3. **P < 0.01 vs. scramble group. **D-E.** High Pi treated mouse VSMCs were infected with scramble or SGK3 shRNA (SGK3-sh). Alizarin Red S staining (**D**), and calcium content (**E**) was used to detect calcium deposits in VSMCs. **P < 0.01 vs. scrambled+Pi group. **F-G.** Human VSMCs treated with 20% human serum (CON) or 20% CKD-serum and SGK3-PROTAC1 for 7 days. Representative images of the Alizarin Red S staining showed the mineral deposition in matrix (**F**). Calcium content of the extracellular matrix was given as Mean ± SD (**G**). **P < 0.01, ***P < 0.001 vs. control group; ^##^*P* < 0.01 vs. CKD-serum group. **H-I.** Mouse VSMCs transiently transfected with SGK3-S486D plasmid **(H)** or treated with SGK3-PROTAC1 **(I)**, in presence or absence of 3 mM Pi. The protein levels of SGK3, RUNX2 and BMP2 were measured by western blot. *P < 0.05, **P < 0.01, ***P < 0.001 vs. control group; ^##^*P* < 0.01, ^###^*P* < 0.001 vs. Pi group; ^△△^*P* < 0.01, ^△△△^*P* < 0.001 vs. SGK3-PROTAC1 group.

To further confirm the role of SGK3 in VC, cultured VSMCs were treated with the SGK3 inhibitor, SGK3-PROTAC1 29, together with high Pi for 7 days. Results from both Alizarin Red S staining and quantification of calcium showed that additional treatment with SGK3-PROTAC1 in cultured mouse VSMCs significantly blunted high Pi-induced the calcium deposition increase (Figure 2A-B). In addition, three different lentivirus knockdown sequences of SGK3, named 93, 94, 95, were synthesized, and the knockdown efficacy was detected by RT-PCR and western blotting. SGK3 shRNA 95 presented the highest inhibition effect than the other two sequences (Figure S3A-B), and thus it was chosen for our following experiment. Similarly, the protein expression levels of SGK3 were reduced after transfection with SGK3 shRNA (Figure 2C). High Pi-induced VSMCs calcification was significantly attenuated by SGK3 knockdown (Figure 2D-E). Human VSMCs were exposed to healthy human serum or pooled uremic serum from ND-CKD patients at stages 4 and 5 and treated with or without SGK3-PROTAC1 for 7 days. Both Alizarin Red S staining and calcium content quantification revealed that SGK3-PROTAC1 significantly inhibited CKD-serum-induced VSMCs calcification (Figure 2F-G). To further investigate the involvement of SGK3 in VSMCs phenotype switching, cultured mouse VSMCs were transfected with the eukaryotic expression vector encoding the constitutively activated S486D mutant of mouse SGK3 (SGK3-S486D) or the control plasmid and then treated with high phosphate. We found that the protein expression levels of RUNX2 and BMP2 were significantly higher in the Pi^+^/S486D^+^ group than in the Pi^+^/S486D^-^ group (Figure 2H). Conversely, SGK3 inhibition reversed high phosphate-induced VSMCs osteoblastic phenotype switching (Figure 2I). We also found the decrease expression of RUNX2, BMP2 and α-SMA through SGK3-shRNA lentivirus transfection (Figure S3C). These findings demonstrate that SGK3 inhibition may benefit the onset and development of VC in CKD.

### SGK3 participates in high phosphate-induced Pit-1 expression and activity

**Figure 3:**
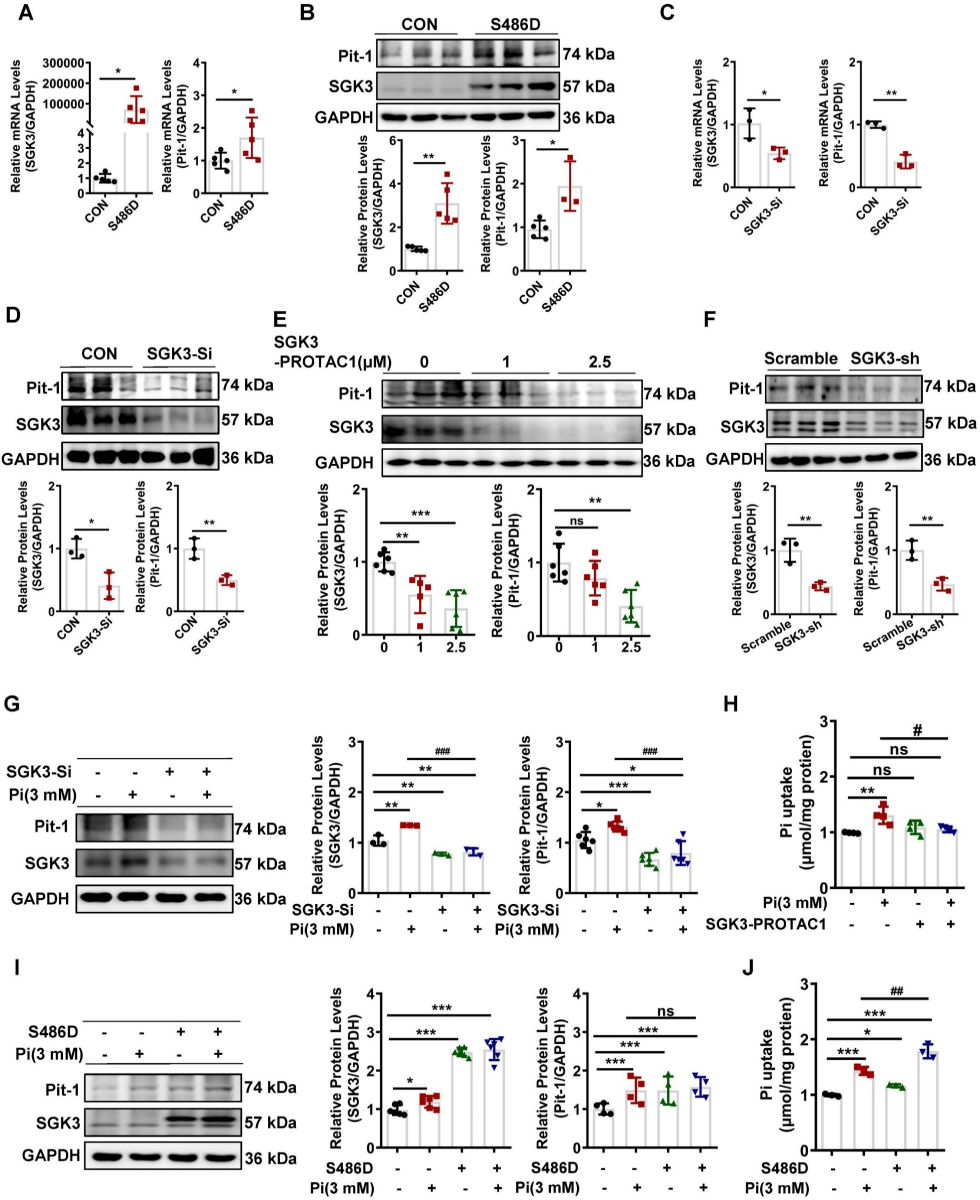
** SGK3 enhanced high phosphate-induced expression of Pit-1 in cultured VSMCs. A-D.** Mouse VSMCs transiently transfected with SGK3-S486D plasmid or SGK3 siRNA. The transcription levels of Pit-1 and SGK3 measured by qPCR **(A, C)**. The protein levels of Pit-1 and SGK3 were measured by western blot **(B, D)**. **E.** Mouse VSMCs treated with SGK3-PROTAC1 for 24 h. Immunoblot of Pit-1 and SGK3 cleavage of cell lysates. **F.** Mouse VSMCs were transfected with scramble or SGK3 shRNA for 72 h. The protein levels of Pit-1 and SGK3 were measured by western blot. *P < 0.05, **P < 0.01, ***P < 0.001 vs. control group. **G-J.** Mouse VSMCs transiently transfected with SGK3 siRNA or SGK3-S486D plasmid, in presence or absence of 3 mM Pi. The protein levels of Pit-1 and SGK3 were measured by western blot **(G, I)**. Pi uptake levels were determined from cell lysates by Pi Assay Kit **(H, J)**. *P < 0.05, **P < 0.01, ***P < 0.001 vs. control group; ^#^*P* < 0.05, ^##^*P* < 0.01, ^###^*P* < 0.001 vs. Pi group.

We further explored the detailed mechanism of action of SGK3 in high phosphate-induced VC. SGK3 has been shown to regulate the intestinal inorganic phosphate (Pi) transporter NaPi2b 16, and Pit-1 is the major phosphate transporter that plays a key role in high phosphate-induced osteoblastic differentiation of VSMCs 30. Next, we explored whether SGK3 directly affects Pit-1. To do this, we constructed SGK3-S486D and SGK3-specific siRNAs (SGK3-Si) to upregulate or downregulate the activity of SGK3 in cultured VSMCs. Compared to the control plasmid transfection group, SGK3-S486D transfection increased the mRNA and protein expression levels of Pit-1 (Figure 3A-B). In addition, we treated VSMCs with different concentration (1 μM and 2.5 μM) of SGK3-PROTAC1 for 24 h (Figure 3E) and found the inhibition efficacy was higher in 2.5 μM group than in 1 μM group. Then, we treated VSMCs with 2.5 μM SGK3-PROTAC1 for 8 h, 12 h, and 24 h respectively and found SGK3 protein expression was maximally inhibited at 24 h (Figure S4A-B). Thus, we chose 2.5 μM SGK3-PROTAC1 stimulated for 24 h for our following experiment. We found that the expression levels of Pit-1 in the SGK3 siRNA, SGK3-PROTAC1 or SGK3 shRNA group were significantly lower than those in the control group (Figure 3C-F). These results suggest that SGK3 is involved in regulating the expression of Pit-1.

Furthermore, to investigate whether SGK3 regulates high phosphate-induced Pit-1 expression and activity, cultured mouse VSMCs were transfected with SGK3-siRNA, or SGK3-S486D, and then treated with high phosphate. We found that high phosphate administration did not increase Pit-1 expression in SGK3-siRNA treated VSMCs (Figure 3G). Similarly, inhibiting SGK3 activation prevented high phosphate-induced phosphate uptake by cultured VSMCs (Figure 3H). In addition, we found that phosphate uptake rather than the protein levels of Pit-1 was significantly increased in the Pi^+^/S486D^+^ group, compared to the Pi^+^/S486D^-^ group (Figure 3I-J), indicating that SGK3-triggered Pit-1 activation was independent on the protein expression levels of Pit-1. Meanwhile, we transiently transfected cultured VSMCs with different qualities (0.5 μg, 1 μg, 2 μg and 4 μg) of SGK3-S486D plasmid for 48 h, and then treated with 2.5 μmol/L SGK3-PROTAC1 for the last 24 h. We also found that, as the dose of SGK3-S486D plasmid increased, the protein levels of calcification markers (RUNX2 and BMP2) and Pit-1 were also gradually increased. Transfection with 1 μg S486D plasmid could totally rescue the inhibition efficacy of 2.5 μmol/L SGK3-PROTAC1 on SGK3 expression (Figure S5A). Thus, we chose 1 μg S486D plasmid and 2.5 μmol/L SGK3-PROTAC1 for phosphate uptake experiment. In line with above results, SGK3 overexpression could reverse SGK3-PROTAC1 induced decrease phosphate uptake in cultured VSMCs (Figure S5B). Overall, these findings suggest that SGK3 inactivation could decrease high phosphate-induced increase protein expression and activity of Pit-1 in VSMCs.

### SGK3 directly binds with Pit-1 at Thr 468 of loop 7 and thus mediated phosphate uptake *in vitro*

**Figure 4:**
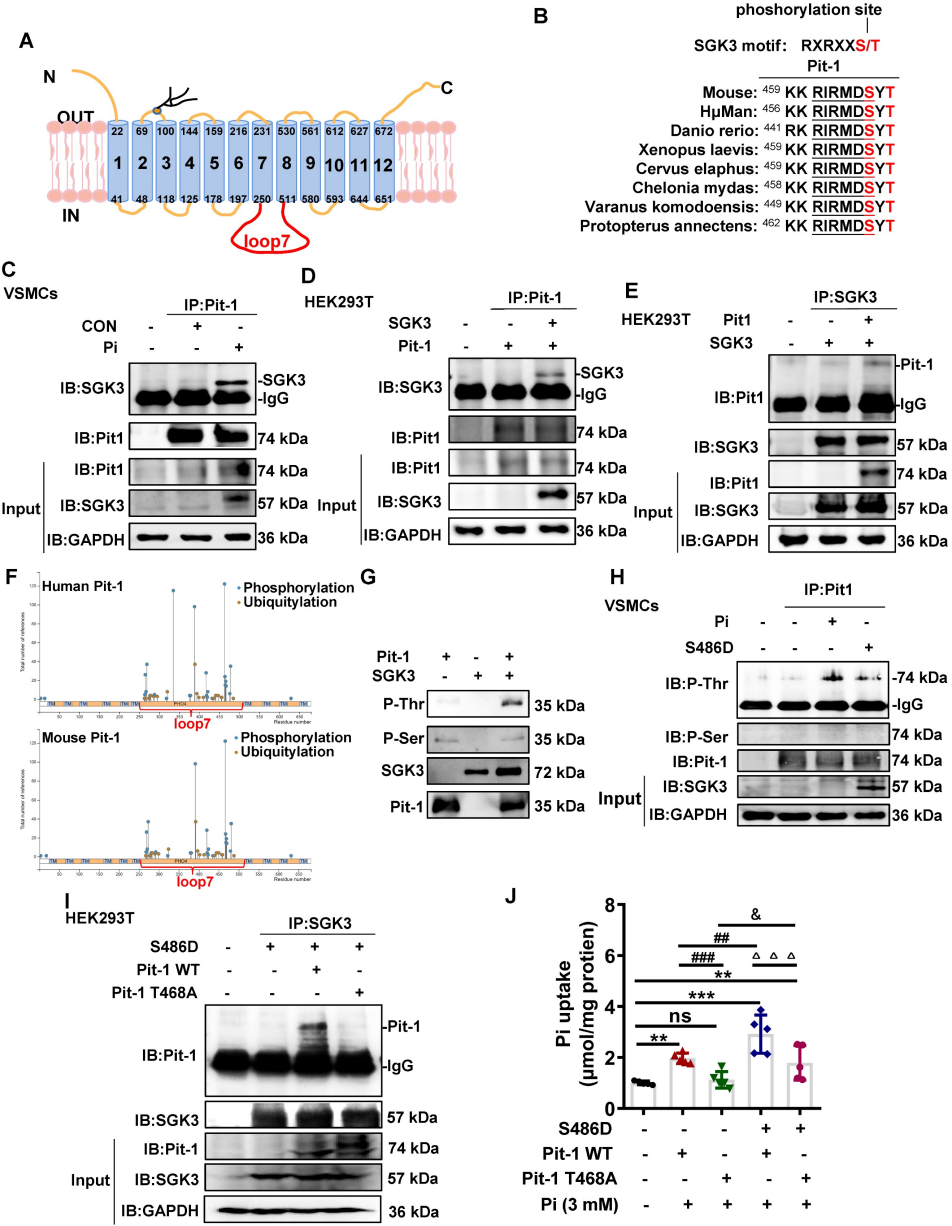
** SGK3 phosphorylates Pit-1 at Thr in loop7. A.** Schematic representation of Pit-1 loop7 domain (residues 251-510). **B.** The putative SGK3 phosphorylation site present in Pit-1 is conserved across different species. **C.** In mouse VSMCs treated with high Pi, whole-cell lysates (WCLs) were precipitated with an anti-Pit-1 antibody as input. The immunoprecipitates were analyzed with a western blot using anti-Pit-1 and anti-SGK3 antibodies. **D-E.** HEK293T cells co-transfected of WT SGK3 and Pit-1 plasmids for 48 h. WCLs were precipitated with the anti-Pit-1 antibody (**D**) or anti-SGK3 antibody (**E**) as input, and the immunoprecipitates obtained with anti-Pit-1 and anti-SGK3 antibodies. **F.** Phosphorylation sites of mouse and human Pit-1 were predicted using phosphosite databases (www.phosphosite.org). **G.** Active SGK3 kinase phosphorylated inactive Pit-1 substrate in vitro, visualized by western blot showing SGK3, Pit-1, Phosphothreonine (P-Thr) and Phosphoserine (P-Ser). **H.** Mouse VSMCs transiently transfected with SGK3-S486D plasmid or 3 mM Pi. WCLs were precipitated with an anti-Pit-1 antibody, and the immunoprecipitates were analyzed with western blot using SGK3, Pit-1, anti-P-Thr and anti-P-Ser antibodies. **I.** HEK293T cells co-transfected with the S686D, Pit-1 wild type or Pit-1 T468A plasmid. A portion of the whole-cell lysate was also subjected to WB analysis as input. **J.** HEK293T cells co-transfected with S686D, Pit-1 wild type or Pit-1 T468A plasmid. Pi uptake levels were determined from cell lysates by Pi Assay Kit. **P < 0.01, ***P < 0.001 vs. control group; ^##^*P* < 0.01, ^###^*P* < 0.001 vs. Pit-1 WT group; ^△△△^*P* < 0.001 vs. S486D+Pit-1 WT group; ^&^*P* < 0.05 vs. Pit-1 T468A group.

As a transmembrane protein, Pit-1 consists of 12 transmembrane domains (TMDs) and a large central intracellular loop 7 (Figure 4A). Given that SGK3 regulates the activity of Pit-1, it is important to note that Pit-1 contains a highly conserved SGK3-phosphorylated motif, RXRXXS/T located in loop7, which is present in multiple species (Figure 4B). Therefore, we performed additional experiments to examine whether SGK3 interacted with and phosphorylate Pit-1. First, mouse VSMCs were cultured *in vitro* and treated with high Pi concentrations, after which cell lysates were subjected to co-IP. Our results demonstrated that the interaction between Pit-1 and SGK3 was increased under high Pi stimulation (Figure 4C). These observations inspired us to determine whether SGK3 binds to Pit-1. To address this question, we co-expressed plasmids containing Pit-1 and SGK3 in HEK293T cells and performed co-immunoprecipitation assays revealed an interaction between Pit-1 and SGK3 (Figure 4D-E). Using the phosphosite database (www.phosphosite.org), we found that most of the phosphorylation sites of human and mouse Pit-1 were located in loop7, including 1 Cys (264), 15 Ser (265, 267, 269, 277, 288, 318, 335, 417, 420, 432, 455, 463, 466, 476, 478), 7 Tyr (376, 378, 388, 418, 421, 464, 467), and 1 Thr (465) of human Pit-1 and 1 Aln (292), 12 Ser (268, 269, 271, 273, 322, 420, 423, 435, 466, 469, 479, 481), 7 Tyr (379, 381, 392, 421, 424, 467, 470) and 2 Thr (458, 468) of mouse Pit-1 (Figure 4F). Therefore, we performed *in vitro* kinase experiments using SGK3 active kinase and purified Pit-1 loop7 proteins, which were expressed in *E. coli* strain BL28. As shown in Figure 4G, SGK3 directly phosphorylates Pit -1 at Thr rather than at Ser. Consistently, we found both high phosphate and SGK3-S486 transfection could promote the phosphorylation of Thr rather than Ser on Pit-1 (Figure 4H), suggesting that Thr (465) of human Pit-1 and Thr (458, 468) of mouse Pit-1 might be the target amino acids of SGK3. Since both human Pit-1 Thr (465) and mouse Pit-1 Thr (468) are located in the conserved sequence (RXRXXS/T), we presumed that the Thr 468 of Pit-1 could be phosphorylated by SGK3. Therefore, we constructed a eukaryotic expression vector encoding the T468A mutant of mouse Pit-1 (Pit-1 T468A). The result revealed that the interaction between SGK3 and Pit-1 disappeared after transfection with Pit-1 T468A, as compared to the Pit-1 WT group (Figure 4I). Furthermore, regardless of SGK3 activation, phosphate uptake decreased after Pit-1 T468A plasmid transfection compared to that in the Pit-1 WT plasmid transfection group (Figure 4J). These results indicate that Thr468 in loop 7 is necessary for SGK3 interaction with Pit-1 and for SGK3-mediated phosphate uptake. Yet, whether the interaction of SGK3 and Pit-1 are phosphorylation-dependent is still need to be further determined.

### SGK3/NF-κB signaling pathway regulates mRNA transcription of Pit-1 in mouse VSMCs

**Figure 5:**
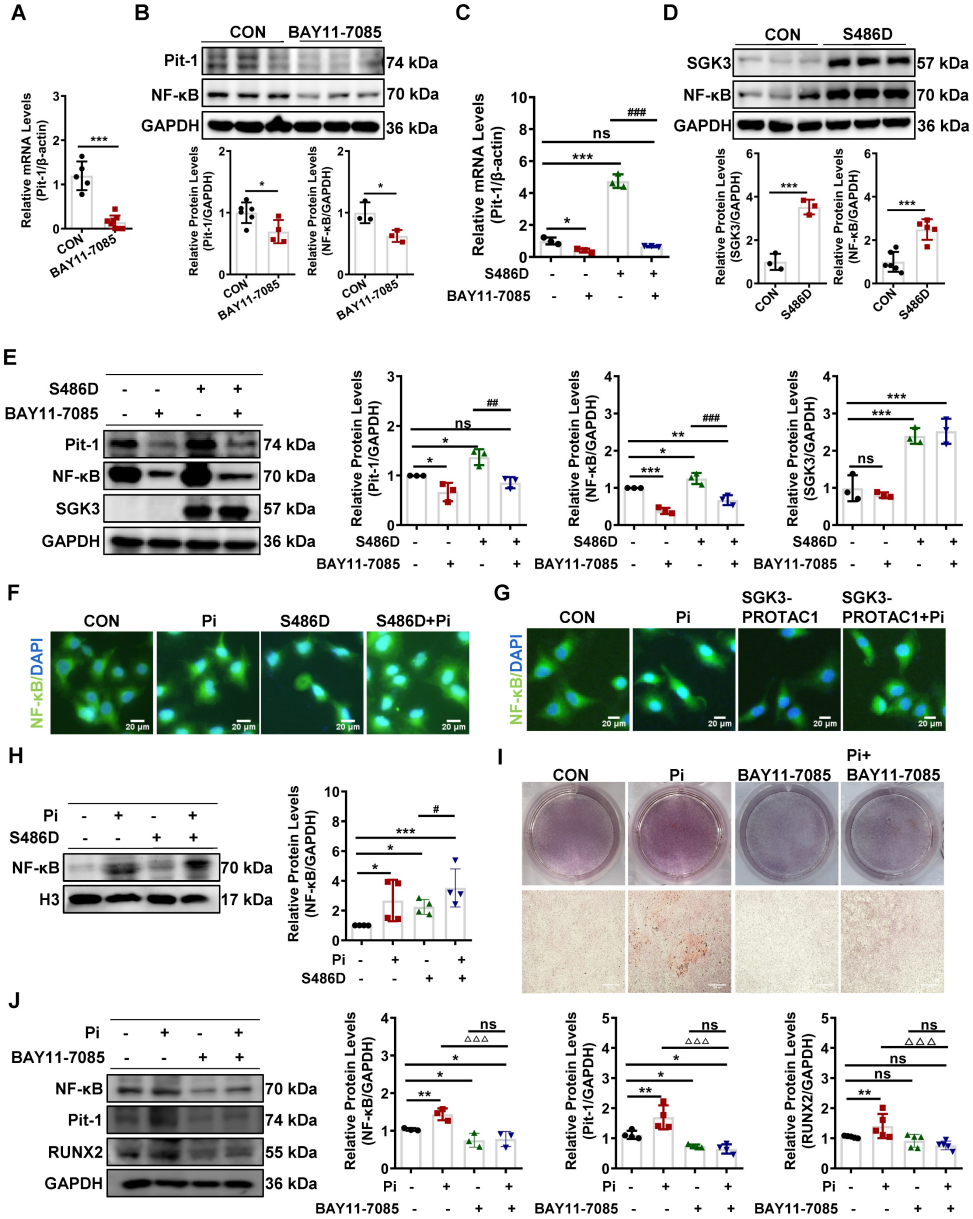
** SGK3/NF-κB signaling pathway regulated the nuclear transcription of Pit-1 in mouse VSMCs.** Mouse VSMCs treated with 20 µmol/L NF-κB inhibitor BAY11-7085. **A.** The mRNA levels of Pit-1 measured by qPCR. **B.** The protein levels of Pit-1 and NF-κB were measured by western blot. **C.** Mouse VSMCs transiently transfected with SGK3-S486D plasmid and then treated with or without 20 µmol/L BAY11-7085. The mRNA levels of Pit-1 measured by qPCR. **D-E.** Mouse VSMCs transiently transfected with SGK3-S486D plasmid **(D)** for 48 h, in presence or absence of BAY11-7085 **(E)**. The protein levels of NF-κB, Pit-1 and SGK3 were measured by western blot. *P < 0.05, **P < 0.01, ***P < 0.001 vs. control group; ^##^*P* < 0.01, ^###^*P* < 0.001 vs. S486D group. **F-G.** Mouse VSMCs transiently transfected with SGK3-S486D plasmid **(F)**, or treated with SGK3-PROTAC1 **(G)**, in presence or absence of high Pi for 24 h. Representative immunofluorescence microscopy images showed NF-κB protein expression and localization in mouse VSMCs. Green labeling, NF-κB expression; blue labeling, nuclei. Scale bars: 20 μm. **H.** Mouse VSMCs transiently transfected with SGK3-S486D plasmid, in presence or absence of Pi for 48 h. The nuclear protein levels of NF-κB was measured by western blot. *P < 0.05, ***P < 0.001 vs. control group; ^#^*P* < 0.05 vs. S486D group. **I.** Mouse VSMCs treated with 3 mM Pi and without or with additional treatment with 20 µmol/L NF-κB inhibitor BAY11-7085 for 7 days. Representative original images showing Alizarin Red S staining. **J.** Mouse VSMCs were treated with BAY11-7085 for 24 h, in presence or absence of high Pi. The protein levels of NF-κB, Pit-1 and RUNX2 were measured by western blot. *P < 0.05, **P < 0.01 vs. control group; ^△△△^*P* < 0.001 vs. Pi group.

To determine the regulatory mechanism underlying activation of SGK3 in promoting the transcription levels of Pit-1, we examined the transcription factor NF-κB, which is known to regulate Pit-1 transcription and is reported to be a downstream target of the SGK protein family, including SGK1 14, 31. In consistent with previous reports, we found NF-κB inhibitor (BAY11-7085) could significantly inhibit the mRNA and protein expression levels of Pit-1 (Figure 5A-B). In addition, BAY11-7085 administration prevented the SGK3 activation-induced increase mRNA expression of Pit-1 (Figure 5C). Furthermore, similar to SGK1, WB distributed that SGK3 activation could further enhance the protein expression of NF-κB (Figure 5D). Furthermore, BAY11-7085 treatment completely alleviates the increase in Pit-1 induced by SGK3 activation (Figure 5E). Immunofluorescence and western blot results showed that compared with the control group, high phosphate could significantly increase the nuclear translocation and nuclear protein levels of NF-κB, and overexpression of SGK3 could further promote the nuclear translocation of NF-κB (Figure 5F, 5H). Moreover, mouse VSMCs were treated with SGK3-PROTAC1 followed by high-phosphate treatment. The immunofluorescence study detected that SGK3-PROTAC1 could partially reverse the high phosphate-induced increase in nuclear translocation of NF-κB (Figure 5G). In addition, results from both Alizarin Red S staining and western blot showed that additional treatment with NF-κB inhibitor (BAY11-7085) in cultured mouse VSMCs significantly blunted high phosphate-induced VSMCs calcification as well as protein expression levels of the osteogenic markers RUNX2 (Figure 5I-J). These observations indicate that SGK3 triggers Pit-1 mRNA transcription and VC by regulating the translocation and the protein expression of NF-κB.

### SGK3 triggered ubiquitin-mediated protein degradation of Pit-1 in cultured mouse VSMCs

**Figure 6:**
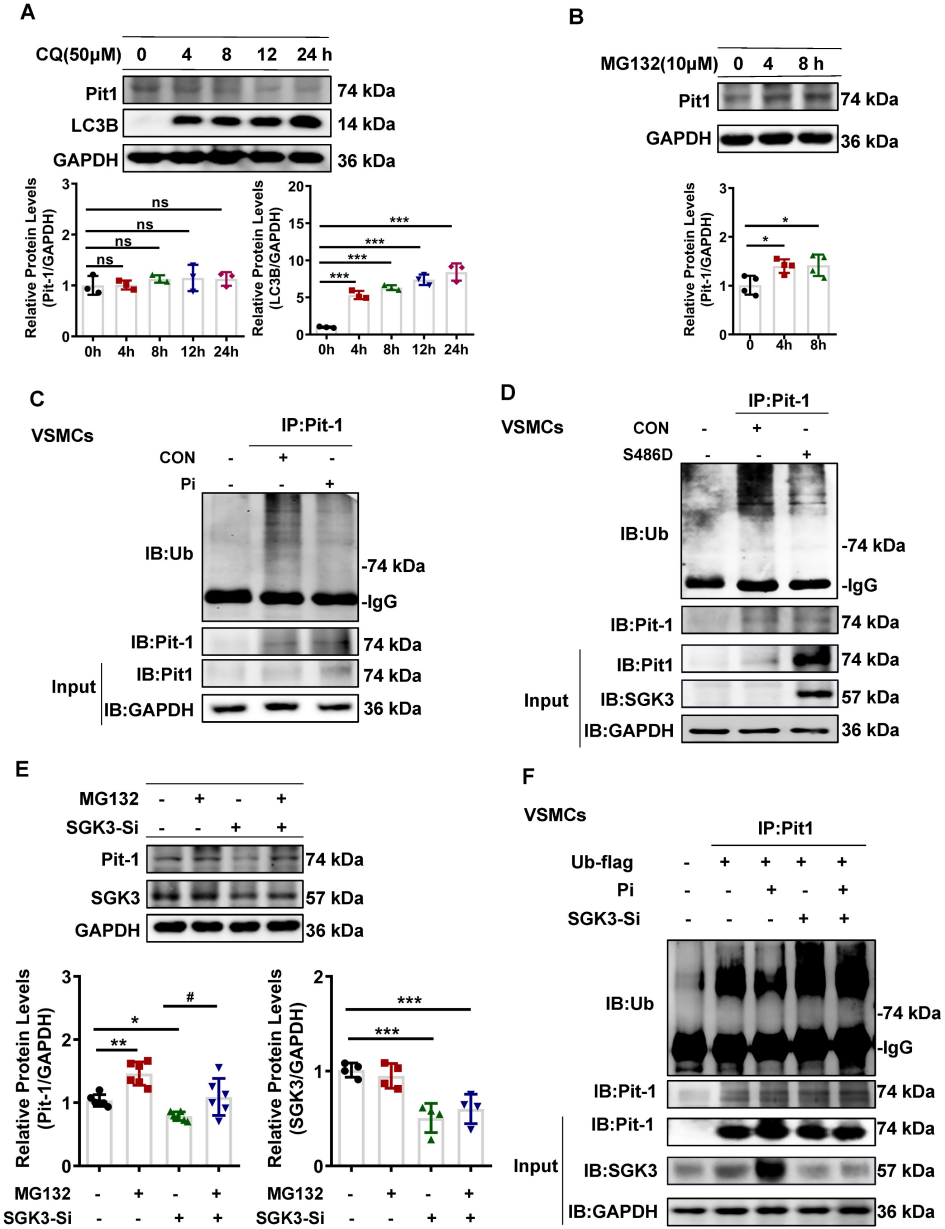
** SGK3 inhibiton partially reversed high phosphate-induced decrease in Pit-1 degradation triggered by the proteasome pathway. A.** Mouse VSMCs treated with 50 μM chloroquine (CQ) for 4 h, 8 h, 12 h, 24 h. The protein levels of Pit-1 and LC3B were measured by western blot. **B.** Mouse VSMCs treated with 10 μM MG132 for 4 h and 8 h. The protein levels of Pit-1 were measured by western blot. *P < 0.05, ***P < 0.001 vs. the control group. **C-D.** Mouse VSMCs treated with 3 mM Pi **(C)** or transfected with SGK3-S486D plasmid **(D)** for 48 h, and MG132 was added for the last 8 h. A portion of the whole-cell lysate was also subjected to WB analysis as input. **E.** Mouse VSMCs transiently transfected with SGK3 siRNA for 48 h and then treated with or without MG132 for the last 8 h. The protein levels of Pit-1 and SGK3 were measured by western blot. *P < 0.05, **P < 0.01, ***P < 0.001 vs. SGK3-Si^-^/MG132^-^ group; ^#^*P* < 0.05 vs. SGK3-Si^+^/MG132^-^ group. **F.** Mouse VSMCs transiently transfected with SGK3 siRNA in presence or absence of 3 mM for 48 h. A portion of the whole-cell lysate was also subjected to WB analysis as input.

To further explore the mechanism of SGK3-mediated Pit-1 protein expression, we examined the role of lysosome- and proteasome-modulated protein in the degradation of Pit-1. We treated it with chloroquine (CQ), a lysosomal inhibitor, or MG132, a proteasome inhibitor. As shown in Figure 6A-B and Figure S6B-C, the protein expression of Pit-1 was significantly increased after MG132 rather than CQ administration, suggesting that the degradation of Pit-1 occurred mainly through the proteasome pathway rather than the lysosome pathway. Consistent with this, the ubiquitin levels of Pit-1 were inhibited in high Pi-treated mouse VSMCs compared to control mouse VSMCs (Figure 6C). Moreover, SGK3 activation prevented the ubiquitin-mediated degradation of Pit-1 (Figure 6D). In addition, MG132 administration reversed the SGK3 siRNA transfection-induced decreased protein expression of pit-1 (Figure 6E), suggesting that SGK3-triggered proteasome-mediated protein degradation of Pit-1. To determine whether SGK3 is involved in the high phosphate-induced decrease in ubiquitinated levels of Pit-1, we performed co-immunoprecipitation studies on mouse VSMCs. Where cells were transfected with SGK3-specific siRNA (SGK3-Si) and treated with high phosphate. The result revealed that the ubiquitin levels of Pit-1 was increased in the Pi^+^/SGK3-Si^+^ group than in the Pi^+^/SGK3-Si^-^ group(Figure 6F). These findings demonstrate that SGK3 inactivation prevents the high phosphate-induced decrease in the ubiquitin levels of Pit-1.

### SGK3/Nedd4-2 signaling pathway modulated Pit-1 protein degradation through the ubiquitin-proteasome pathway

**Figure 7:**
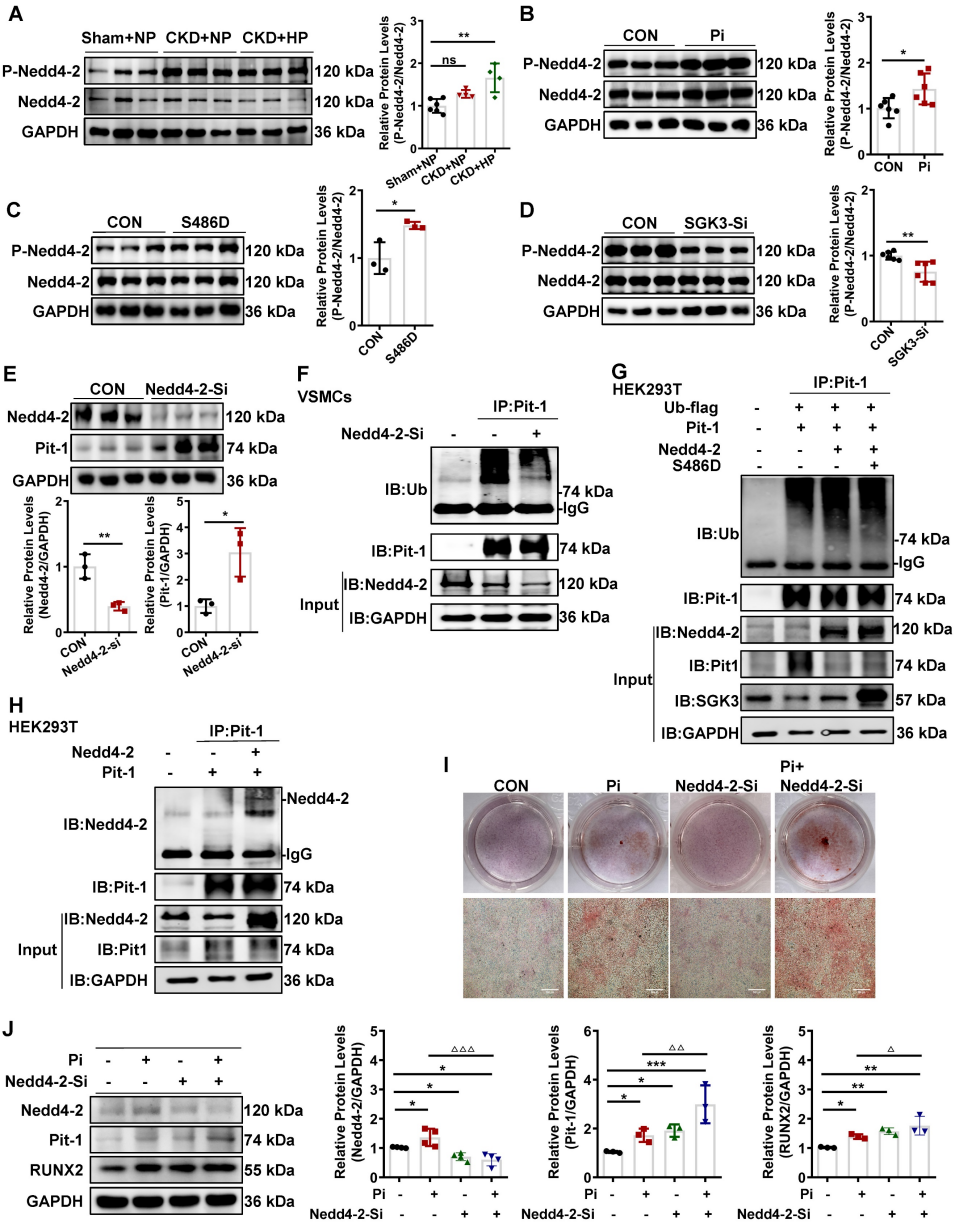
** SGK3/Nedd4-2 signaling pathway prevents ubiquitin-mediated degradation of Pit-1.** Mice were modeled with CKD by 5/6 nephrectomy and fed high phosphate diet.** A.** The protein levels of phospho-Nedd4-2 (P-Nedd4-2) and Nedd4-2 in the aorta were measured by western blot. **P < 0.01, vs. Sham+NP group. **B-D.** Mouse VSMCs treated with 3 mM Pi **(B)**, or transfected with SGK3-S486D plasmid **(C)** and SGK3 siRNA** (D)** for 48 h. The protein levels of P-Nedd4-2 and Nedd4-2 were measured by western blot.** E.** Mouse VSMCs transiently transfected with Nedd4-2 siRNA for 48 h. The protein levels of Pit-1 and Nedd4-2 were measured by western blot. *P < 0.05, **P < 0.01 vs. the control group. **F.** Mouse VSMCs transiently transfected with Nedd4-2 siRNA for 48 h and followed by MG132 treatment for the last 8 h. Ubiquitination assay were precipitated with an anti-Pit-1 antibody, and the immunoprecipitates were analyzed with western blot using anti-Pit-1 and anti-ubiquitin (Ub) antibodies. **G-H.** The HEK293T cells were transfected with the indicated plasmids for 48 h. Ubiquitination assay **(G)** and Co-IP analysis **(H)** were precipitated with an anti-Pit-1 antibody and the immunoprecipitates were blotted with anti-Nedd4-2, anti-Pit-1, anti-ubiquitin (Ub), anti-SGK3 and anti-GAPDH antibodies. I. Mouse VSMCs were transfected with Nedd4-2 siRNA and without or with additional treatment 3 mM Pi for 7 days. Representative original images showing Alizarin Red S staining. J. Mouse VSMCs transiently transfected with Nedd4-2 siRNA for 48 h, in presence or absence of high Pi. The protein levels of Nedd4-2, Pit-1 and RUNX2 were measured by western blot. *P < 0.05, **P < 0.01, ***P < 0.001 vs. control group; ^△^*P* < 0.05, ^△△^*P* < 0.01, ^△△△^*P* < 0.001 vs. Pi group.

We previously reported that the SGK3/Nedd4-2 signaling pathway is involved in the pathogenesis of CKD by regulating the degradation of nephrin and ezrin through ubiquitin-proteasome-mediated protein degradation 32. To explore how SGK3 specifically regulates the degradation of Pit-1. First, we first examined whether the SGK3 target protein Nedd4-2, a common E3 ubiquitin ligase 33, was involved in SGK3 triggered ubiquitin-mediated protein degradation of Pit-1. Our findings confirmed that the p-Nedd4-2/Nedd4-2 ratio was significantly increased in CKD mice's calcified aorta and in high Pi-treated VSMCs *in vitro* (Figure 7A-B). Furthermore, we confirmed that SGK3 activation enhanced Nedd4-2 phosphorylation in cultured VSMCs. This indicates that the p-Nedd4-2/Nedd4-2 ratio increased significantly in SGK3-S486D plasmid-transfected VSMCs and decreased dramatically in SGK3 knocked-down VSMCs (Figure 7C-D), indicating the involvement of the SGK3/Nedd4-2 signaling pathway in high Pi-induced VSMCs calcification.

To further elucidate whether Nedd4-2 regulates protein expression and ubiquitin levels of Pit-1. First, we transfected mouse VSMCs with Nedd4-2-siRNA (Nedd4-2-Si) to knock down the activity of Nedd4-2. The result shows that the protein expression levels of Pit-1 were upregulated, and the ubiquitin levels of Pit-1 were downregulated after Nedd4-2 knockdown (Figure 7E-F). Consistent with those of mouse VSMCs, we also detected the similar effect of Nedd4-2 siRNA on Pit-1 expression and the same degradation pathway of Pit-1 in cultured human VSMCs (Figure S6D). Together, our results strongly suggest that the inhibition of Nedd4-2 prevents the ubiquitin-mediated degradation of Pit-1. Next, we tested whether SGK3 regulated the activity of Nedd4-2 and affected the ubiquitin levels of Pit-1. We performed co-IP experiments on HEK293T cells to verify this. Cells were transfected with Pit-1, Ub-flag, Nedd4-2, with or without the plasmid SGK3-S486D. In the Pit-1 and Nedd4-2 co-transfected group, the ubiquitin levels of Pit-1 increased, whereas those of Pit-1 decreased after co-transfection with the SGK3-S486D plasmid (Figure 7G). As mouse Pit-1 harbors PY, a motif targeted by Nedd4-2, it was not surprising that Nedd4-2 and Pit-1 interacted in HEK293T cells under basic conditions (Figure 7H). In addition, results from both Alizarin Red S staining and western blot showed that additional treatment with Nedd4-2-siRNA in cultured mouse VSMCs further promoted high phosphate-induced VSMCs calcification (Figure 7I-J). Therefore, the inhibition of Nedd4-2 activity mediated by SGK3 activation also downregulates the ubiquitin-mediated protein degradation of Pit-1 and enhances VC.

## Discussion

Our study showed that SGK3 is a decisive regulator of VC induced by high phosphate. Specifically, SGK3 enhances the expression and activity of Pit-1, which promotes VC induced by elevated phosphate in patients with CKD. Mechanistically, SGK3 promotes the transcription of Pit-1 through NF-κB, downregulates ubiquitin levels of Pit-1 by inhibiting the activity of Nedd4-2, and increases Pit-1 activity via interaction with and phosphorylation of Pit-1 at Thr 468 of loop7.

As a serine/threonine protein kinase, SGK3 has diverse functions in different cell types, including tumors, podocytes, and immunocytes. Although mounting evidence suggests that SGK1, an isoform of SGK that shares 80% homology with SGK3, is upregulated in the cardiovascular system under several pathophysiological conditions 14, the role of SGK3 in the vasculature is entirely unknown. In this study, the mRNA and protein expression levels of SGK3 in VSMCs were upregulated following exposure to uremic patient serum, calcification media, and phosphate. Interestingly, increased SGK3 protein expression was also observed and distributed in uremic mice's calcified regions of the aorta. Moreover, SGK3 expression positively correlated with the aortic calcium content. Accordingly, we found that SGK3 protein expression was significantly enhanced in the calcified AVFs of uremic mice. Therefore, these findings suggest that SGK3 is remarkably enhanced in the vasculature after calcifying agent administration and is required for the regulation of VC in CKD.

SGK3 is a decisive regulator of NaPi2b mediated intestinal phosphate transport, and has previously been shown to maintain phosphate homeostasis 16. Accordingly, the current observations indicate that SGK3 is a powerful modulator of Pit-1, a homology of NaPi2b, and thus triggers VSMCs phosphate uptake. In VSMCs, Pit-1 is more abundant than Pit-2 and is required for elevated phosphate-induced RUNX2 expression and activity 34, 35. Unlike intimal atherosclerosis, phosphate-induced medial VC is mainly mediated by osteogenic conversion 36. The subtotal nephrectomy mouse model administered a high-phosphate diet exhibited significantly increased vascular expression of RUNX2 and BMP2, which was in line with previous findings. Therefore, SGK3 may regulate phosphate-induced VC via Pit-1. In support of this notion, upregulation of Pit-1 by SGK3 activation increased the protein expression levels of RUNX2 and BMP2, whereas inhibition of SGK3 blunted high phosphate-induced VSMCs calcification by affecting osteochondrogenic phenotypic switching, indicating that SGK3 could be a key factor in Pit-1 triggered calcification signaling.

VC is recognized as a pathological vascular disorder, and involved many pathways, including the role of inflammatory responses, endoplasmic reticulum stress, mitochondrial dysfunction, iron homeostasis, metabolic imbalance 37. Mounting evidence has shown that the transcription factor NF-κB plays a crucial role in VC and is activated in patients with CKD 38. Consistent with these findings, we observed that high phosphate induces a sustained nuclear distribution of NF-κB in VSMCs. Previous studies have also shown illustrate that NF-κB activation promotes nuclear transcription of Pit-1, BMP2, and Runx2 39. Voelkl et al. reported that NF-κB activation was required for VSMCs calcification, which is mediated by SGK1, one of SGK3 isoform 14, 40. However, it is unclear whether SGK3 can regulate NF-κB activation. In this study, we confirmed that similar to SGK1, SGK3 activation promoted the nuclear translocation of NF-κB and thus enhanced Pit-1 nuclear transcription.

Pit-1 is a transmembrane protein that contains 12 transmembrane domains (TMDs) and a large central intracellular loop 7 whose functions remain unclear 39, 40. Sun et al. demonstrated that the loop7 domain of Pit-2 could be phosphorylated by AKT thus regulating plasma membrane localization and phosphate uptake 41. Although the loop7 domain of Pit-1 shares 56% homology with Pit-2, and SGK3 has a similar structure, substrate specificity, and function as AKT 42, whether SGK3 can phosphorylate Pit-1 at loop7 remains unknown. By analyzing the amino acid sequence of Pit-1, we found that loop7 of Pit-1 contains an RIRMDS motif, a conserved phosphorylation sequence of SGK3. Based on this structure, we verified that SGK3 interact with Pit-1. However, in the traditional phosphorylation sequence, SGK3 directly phosphorylates Pit-1 at Thr rather than Ser and RIRMDS. In addition, we found that the enhanced phosphate uptake induced by SGK3 activation was independent of the increased protein expression of Pit-1 under high phosphate exposure, and the mutation of T468 in loop7 of Pit-1 resulted in a decrease in the interaction of SGK3 with Pit-1, and blunting SGK3 triggered high phosphate-induced elevated phosphate uptake. These results suggest that SGK3 interacts with and phosphorylated Pit-1, increasing its activity, but the exact effect of SGK3 phosphorylated on Pit-1 needs further investigation.

The cellular degradation pathway mainly involves the ubiquitin-proteasome and autophagy-lysosome systems, with the ubiquitin-proteasome system being responsible for more than 80% of cellular protein degradation 43. It has been reported that both the proteasome and the endosomal sorting complexes required for transport (ESCRT)-lysosomal axis can mediate Pit-1 protein degradation in phosphate-replete cells, but ESCRT-dependent Pit-1 degradation decreases during phosphate starvation 44. However, there have been few studies on the degradation of Pit-1 in VSMCs under high phosphate administration. In this study, we found that the expression of Pit-1 was upregulated by proteasome inhibitors rather than lysosomal inhibitors. Our study found that similar to NaPi2b, SGK3 was found to regulates the ubiquitination and degradation of Pit-1 by inhibiting the ubiquitin ligase Nedd4-2 16. The WW domains of Nedd4-2 can bind to the PPxY sequence of substrate proteins, mediating their ubiquitination and degradation of proteins, such as NHE3, ENaC, and NCC 45, 46. In this study, Nedd4-2 was found to interact with Pit-1, which has a PPvY sequence in mouse Pit-1 protein, but not in human Pit-1. Thus, we suggested the need for further investigation into whether Nedd4-2 regulates the ubiquitination of human Pit-1 and the exact ubiquitin site of Nedd4-2 in mouse Pit-1. Additionally, we noted the possibility that Nedd4-2 indirectly regulates the ubiquitination and degradation of human Pit-1.

## Conclusions

In summary, SGK3 is elevated in the VC of patients with CKD; SGK3 increases Pit-1 mRNA and protein expression while reducing its degradation by the ubiquitin-proteasome degradation. Furthermore, SGK3 enhances Pit-1 nuclear transcription through NF-κB, inhibiting ubiquitin-mediated protein degradation of Pit-1 through Nedd4-2, and increases the activity of Pit-1 by interaction with Thr 468 on loop7. Overall, this study demonstrated that SGK3 promotes CKD-associated VC by regulating Pit-1 expression and activity.

## Supplementary Material

Supplementary figures and table.
