# Supplementary material for: SGK3 promotes vascular calcification via Pit-1 in chronic kidney disease
Source: Theranostics. 2024 Jan 1;14(2):861–78. doi: 10.7150/thno.87317 (PMC10758069; doi:10.7150/thno.87317)
Supplement: Supplementary file 1 — Supplementary figures and table. [file thnov14p0861s1.pdf]

Supplementary materials

Table S1 Characteristics and biochemical parameters of healthy (control) and uremic serum.

	Control serum	Uremic serum
Creatinine (μmol/L)	67.2 ± 13.6	946.83 ± 307.34
Urea nitrogen (mmol/L)	3.55 ± 1.00	31.48 ± 10.89
Serum calcium (mmol/L)	2.45 ± 0.11	1.91 ± 0.38
Serum phospate (mmol/L)	1.22 ± 0.15	2.27 ± 0.62

Biochemical parameters are given as average ± SE in the indicated units.

Supplemental Fig. 1

A

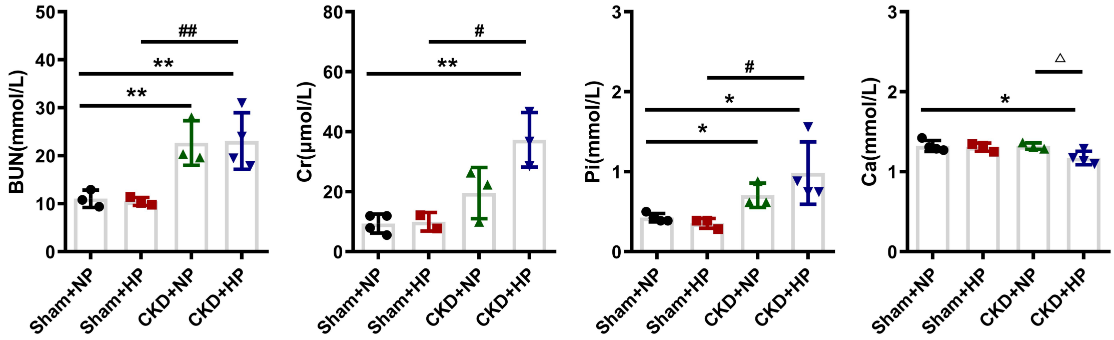

B

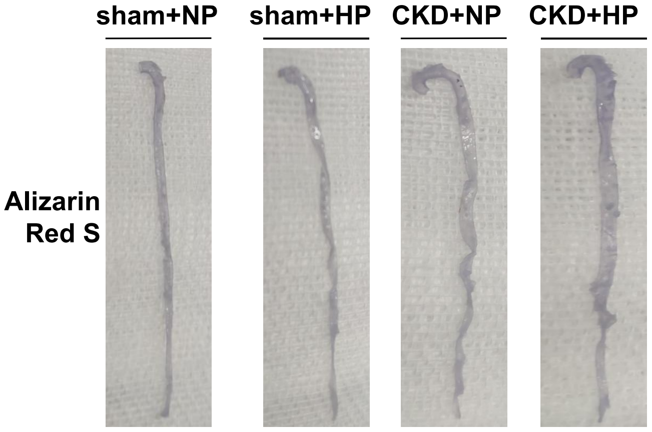

C

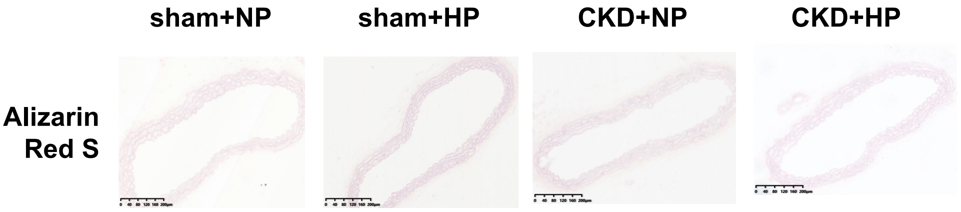

Supplemental Fig. 2

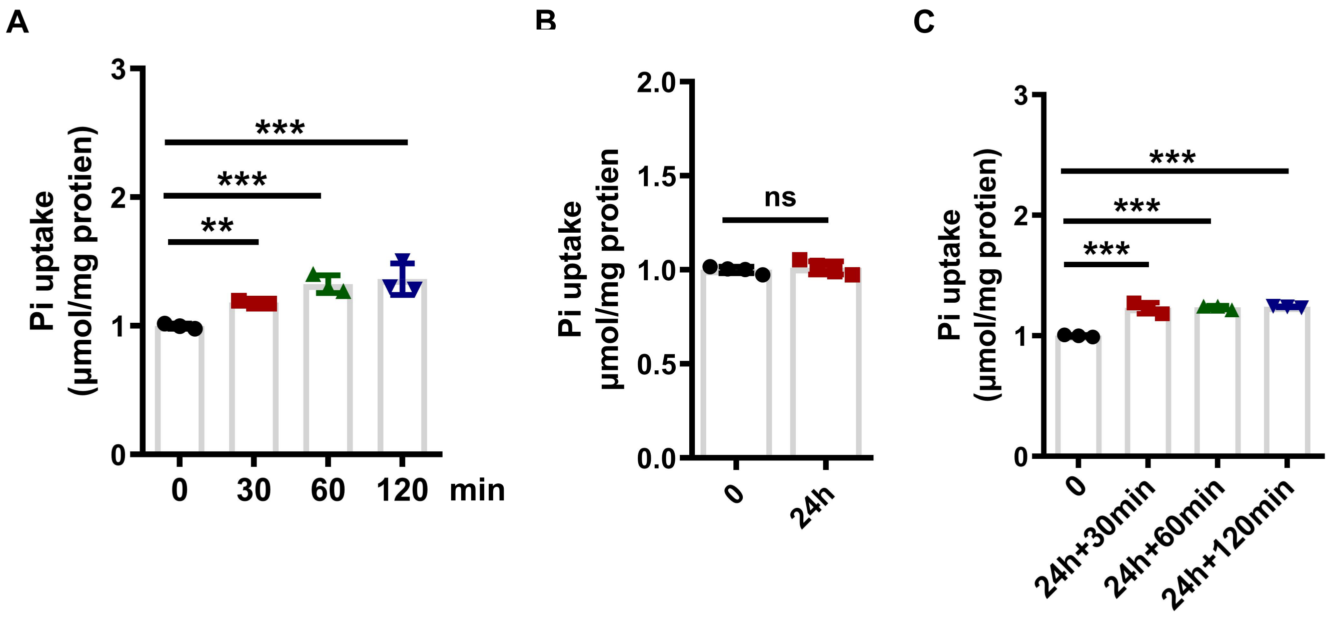

Supplemental Fig. 3

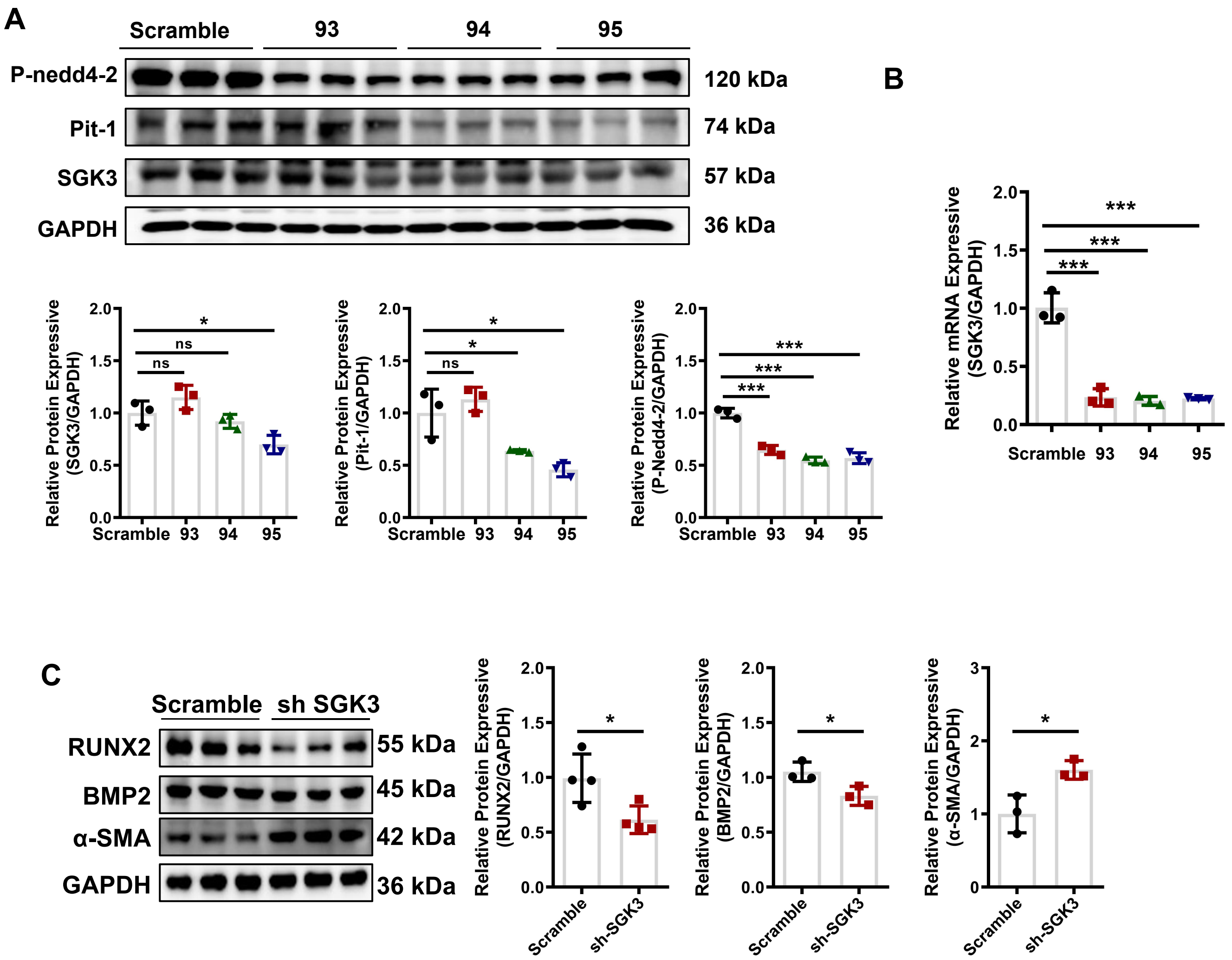

Supplemental Fig. 4

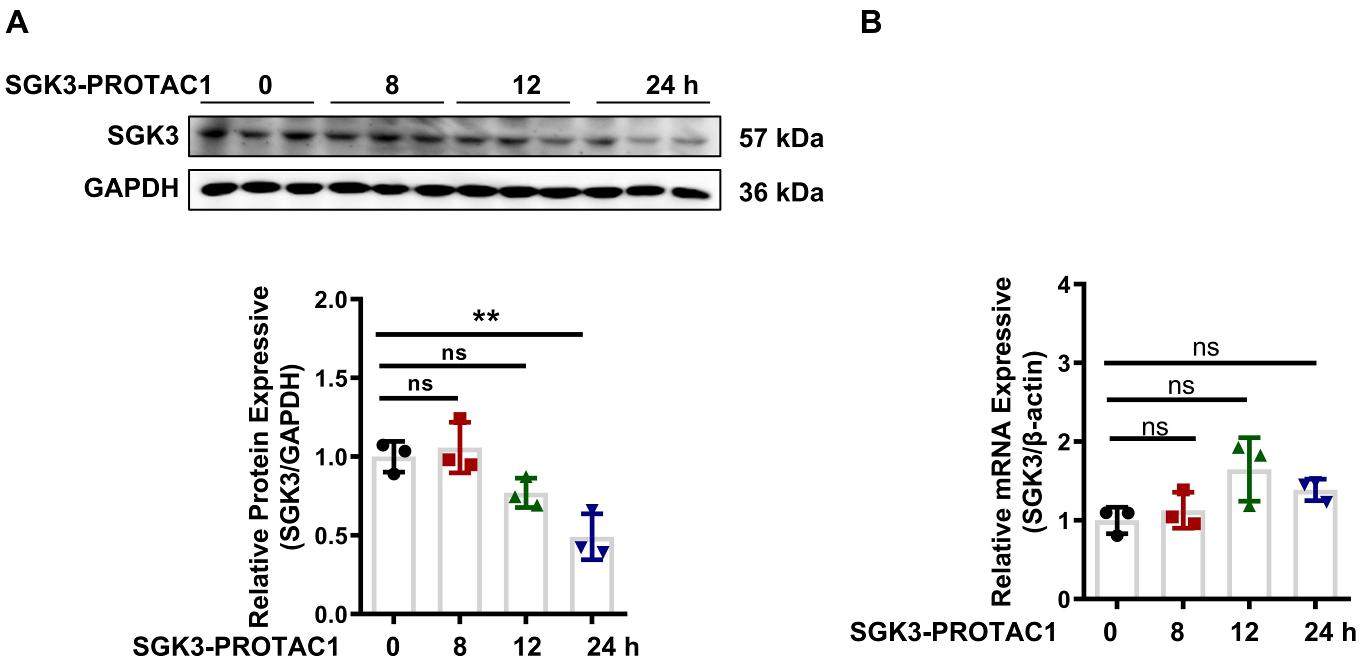

Supplemental Fig.5

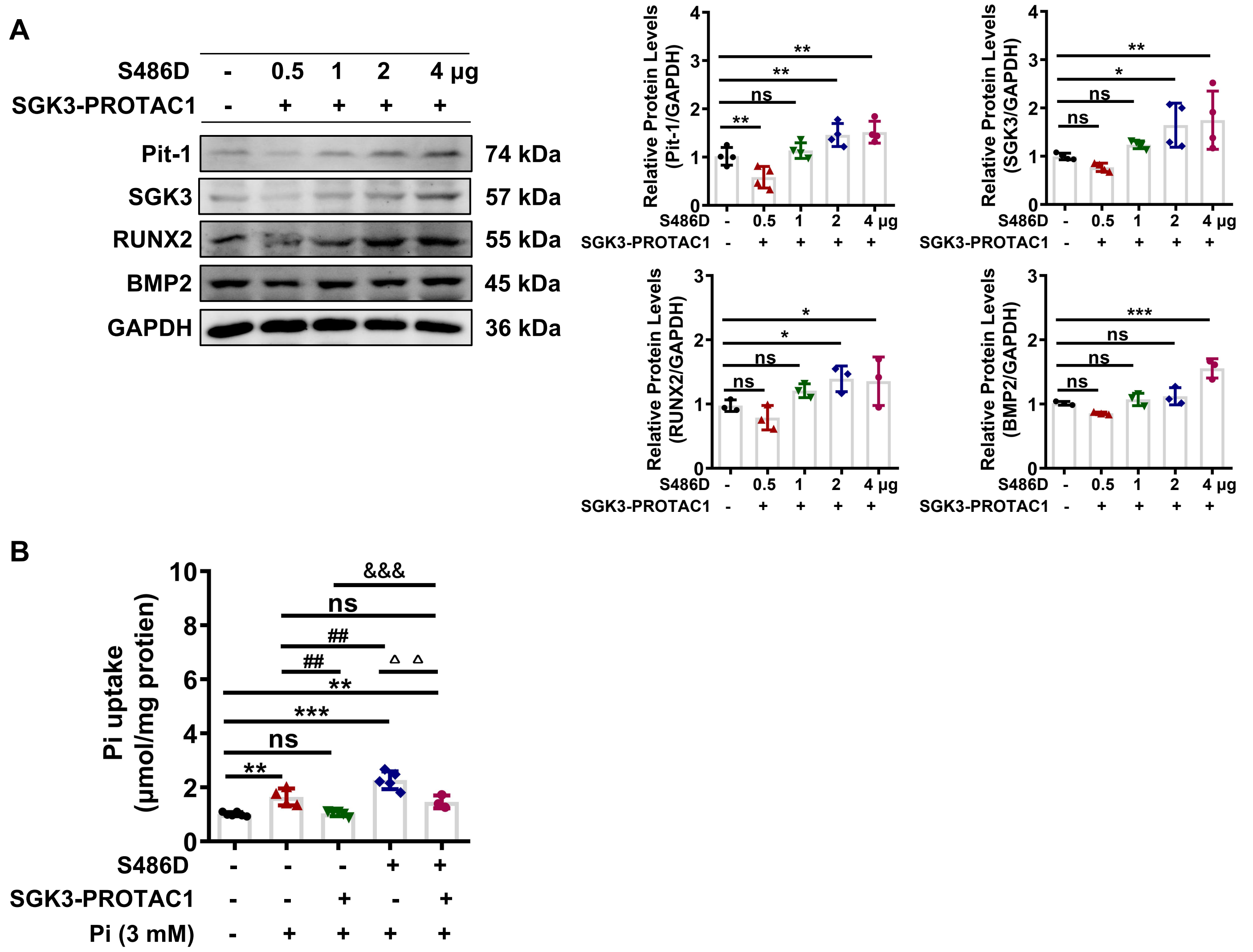

Supplemental Fig.6

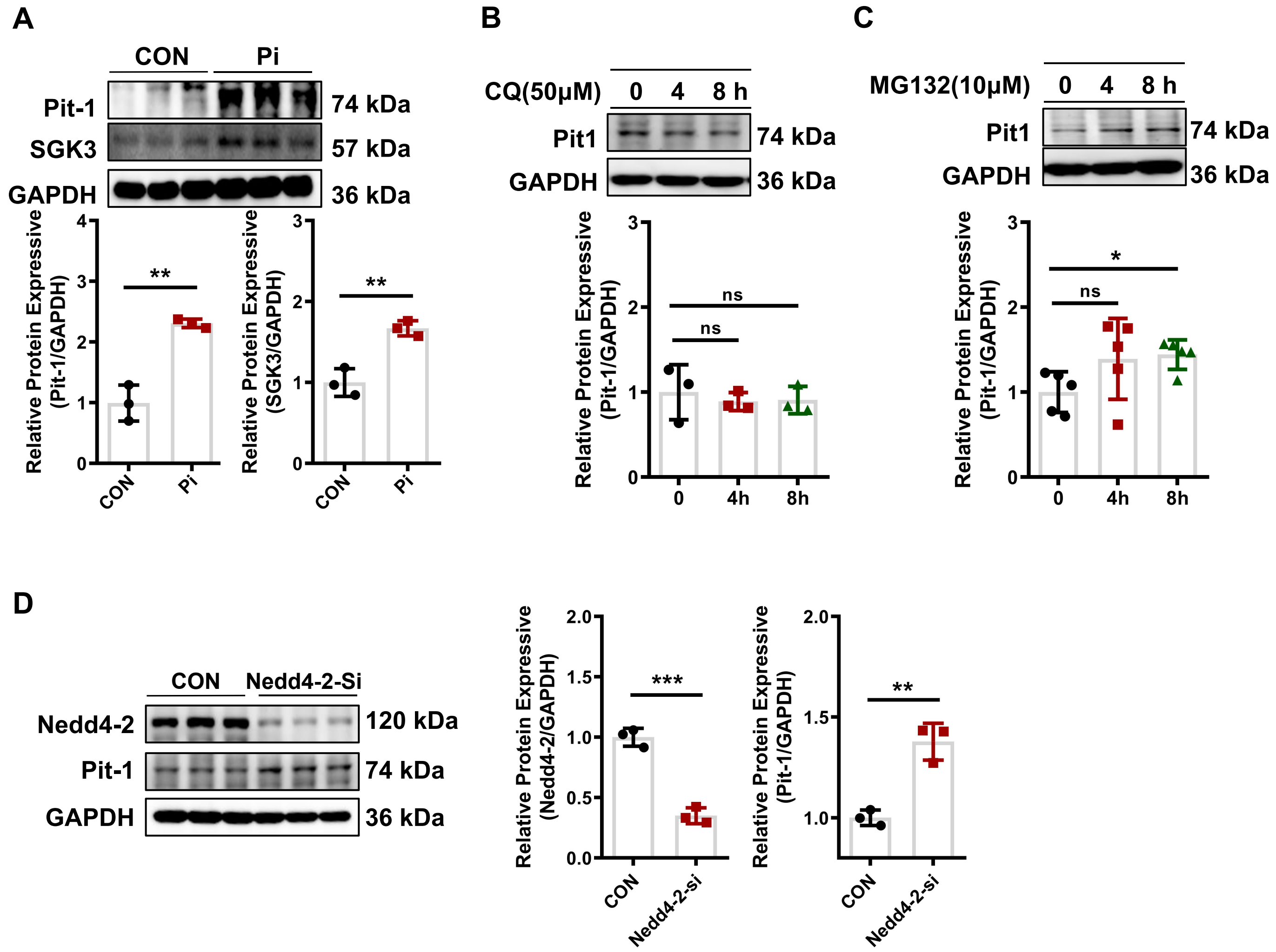

Supplementary figure legends

Supplemental Figure 1. There is not vascular calcification in C57BL/6J mice. Male C57BL/6J mice modeled with CKD by 5/6 nephrectomy and fed with a high-phosphate diet for 8 weeks after surgery. **A.** At the experimental end point, blood urea nitrogen (BUN), serum creatinine (Cr), calcium (Ca), and phosphate (Pi) were detected. **B, C.** Alizarin red S staining, representative staining of Alizarin red S was detected in the aorta of CKD mice.

Supplemental Figure 2. The phosphate uptake at different times in VSMCs. A. Mouse VSMCs treated with 3 mM Pi for 30 min, 60 min, 120 min, Pi levels were determined from cell lysates by Pi Assay Kit. **B.** Mouse VSMCs treated with 3 mM Pi for 24 h, Pi levels were determined from cell lysates by Pi Assay Kit. ns vs. control group. **C.** Mouse VSMCs treated with 3 mM Pi for 24h, and then restimulation for 30 min, 60 min, 120 min after elution, Pi levels were determined from cell lysates by Pi Assay Kit. **$P < 0.01$, ***$P < 0.001$ vs. control group.

Supplemental Figure 3. Inhibition of SGK3 attenuated the expression of Pit-1 and phenotype switching in vitro. A. Mouse VSMCs were infected with scramble or three different lentiviruses knockdown sequences of SGK3 named 93, 94, 95 for 72 h. Western blot analysis of SGK3, Pit-1 and P-Nedd4-2. **B.** Mouse VSMCs were infected with scramble or SGK3 shRNA 95 (sh-SGK3) for 7 d. Western blot analysis of SGK3 BMP2, Runx2, α-SMA and Pit-1. *$P < 0.05$, **$P < 0.01$, ***$P < 0.001$ vs. scramble group.

Supplemental Figure 4. Transcription level as well as protein level of SGK3 after treated with SGK3-PROTAC1. A, B. Mouse VSMCs treated with 2.5 μmol/L SGK3-PROTAC1 for 8 h, 12 h, 24 h. Immunoblot of SGK3 cleavage of cell lysates **(A)**. The transcription levels of SGK3 measured by qPCR **(B)**. **$P < 0.01$ vs. control group.

Supplemental Figure 5. The calcification change and phosphate uptake of Overexpression of SGK3 after treated with SGK3-PROTAC1. A. Mouse VSMCs transiently transfected with SGK3-S486D plasmid (0.5 μg, 1 μg, 2 μg, 4 μg) for 48 h, and treated with 2.5 μmol/L SGK3-PROTAC1 for 24 h. Western blot and related semi-quantificated analysis of SGK3, Pit-1, RUNX2 and BMP2. **B.** Mouse VSMCs transiently transfected with S686D plasmid (1 μg) for 48 h, treated with 2.5 μmol/L SGK3-PROTAC1 for 24 h and 3 mM Pi for 2 h. Pi levels were determined from cell lysates by Pi Assay Kit. **P < 0.01, ***P < 0.001 vs. control group; $^{\#\#}$P < 0.01 vs. Pi group; $^{\triangle\triangle}$P < 0.01 vs. S486D+Pi group; $^{\&\&\&}$P < 0.001 vs. SGK3-PROTAC1+Pi group.

Supplemental Figure 6. The expression levels of Pit-1 in HaSMCs. A. Human VSMCs treated with 3 mM Pi for 7 days. Western blot and related semi-quantificated analysis of SGK3 and Pit-1. **B.** Human VSMCs treated with 50 μM chloroquine (CQ) for 4 and 8 h. The protein levels of Pit-1 were measured by western blot. **C.** Human VSMCs treated with 10 μM MG132 for 4 and 8 h. The protein levels of Pit-1 were measured by western blot. **D.** Human VSMCs transiently transfected with Nedd4-2 si-RNA for 48 h. The protein levels of Pit-1and Nedd4-2 were measured by western blot. **P* < 0.05, ***P* < 0.01, ****P* < 0.001 vs. the control group.
